# Tribological properties of hydroxyapatite-coated nanorods on Ti-6Al-4V surfaces

**DOI:** 10.1038/s41598-025-03253-8

**Published:** 2025-05-31

**Authors:** Sarah Akua Osafo, Tabiri Asumadu, Desmond Klenam, Precious Etinosa, John David Obayemi, Benjamin Agyei-Tuffour, Abu Yaya, David Dodoo-Arhin, Stanley Chijioke Eluu, Wole Soboyejo

**Affiliations:** 1 Department of Materials Science and Engineering, School of Engineering Sciences, College of Basic and Applied Sciences, University of Ghana, Legon, Accra Ghana; 2 Department of Biomaterial Sciences, Dental School, University of Ghana, Korle Bu Campus, Accra, Ghana; 3 Department of Mechanical Engineering, Program in Materials Science and Engineering, Worcester Polytechnic Institute, 100 Institute Road, Worcester, MA 01609 USA; 4 Department of Materials Engineering, Sunyani Technical University, Sunyani, Ghana; 5 Global Center of Advanced Materials and Manufacturing, Department of Mechanical Engineering, College of Engineering, State University of New York (SUNY) Polytechnic Institute, 100 Seymour Street, Utica, NY 13502 USA; 6 Next Frontiers in Advanced Material Laboratory, School of Chemical and Metallurgical Engineering, University of the Witwatersrand, 1 Jan Smuts Avenue, Johannesburg, 2050 South Africa; 7 Department of Biomedical Engineering, Worcester Polytechnic Institute, Gateway Park Life Sciences and Bioengineering Centre, 60 Prescott Street, Worcester, MA 01609 USA; 8 Department of Biotechnology, Ebonyi State University, Abakaliki, Nigeria; 9 Department of Pharmaceutical Microbiology and Biotechnology, Faculty of Pharmaceutical Sciences, Nnamdi Azikiwe University, Awka, Anambra State Nigeria

**Keywords:** Bioactivity, Osseointegration, Surface modification, Titanium alloys, Dental implants, Wear resistance, Implants, Structural properties

## Abstract

This paper presents the tribological properties and bioactivity of nanostructured hydroxyapatite (HA) from biowaste sources and coated onto Ti-6Al-4V substrates using a novel pack cementation method. The process introduced HA pillars/nanorods on the surfaces of Ti-6Al-4V to enhance their osseointegration for dental implants. The mechanical and tribological properties were studied with nanoindentation, and pin-on-disk techniques, following the microstructural characterization of the coatings with atomic force microscopy, X-ray diffraction, Raman spectroscopy, scanning electron microscopy, and energy dispersive x-ray spectroscopy. The study also examines the surface bioactivity and elucidates the underlying friction and wear mechanisms of the HA-coated and annealed Ti-6Al-4V surface. The study results show a bone bonding capacity of the biowaste-derived HA-coated substrate with improved hardness and tribological properties. The implications of the study are discussed for the development of nano-structured HA-coated Ti-6Al-4V for dental implants with improved osseointegration for dental and biomedical applications.

## Introduction

Oral health challenges impact approximately 46% of the global population^1,2^, with ~ 268 million people suffering from edentulism as of 2017^1^. This condition significantly affects chewing efficiency and increases the risk of oral infections, necessitating the use of dental implant^2,3^. Given the growing demand for dental implants, which is now a multibillion-dollar industry, there is a critical need for implant materials to possess superior structural and functional properties for structural integrity and reliability^4–7^. This will significantly reduce the need for revision surgeries and decrease the direct and indirect cost implications.

Titanium alloys have been widely used in biomedical applications. Typically, the Ti-6Al-4V alloys are largely used due to their excellent biocompatibility, corrosion resistance, and mechanical strength^8–11^. However, their bioinert nature limits osteoconductivity, posing challenges for osseointegration^12^. To enhance the bioactivity of Ti-6Al-4V implants, surface modification techniques have been explored, with hydroxyapatite (HA or HAp) coatings emerging as a promising solution^11,13^.

The term “apatite” is part of a group of compounds with molecular formula M_10_(XO_4_)_6_Z_2_^14^. The “M” is the cation and XO_4_^3−^ and Z^−^ are the anions^14^. The general apatite molecular formula is Ca_5_(PO_4_)_3_(OH, F, Cl), where M represents calcium, Z represents the hydroxyl radical, and X represents phosphorus. The molecular formula of HA has a Ca/P ratio of ~ 1.67. As a stoichiometric type of HAp, it is ~ 39% by weight of Ca, ~ 18.5% P and 3.38% OH. Thus, HAp is a calcium phosphate (CaP) ceramic with a molecular formula of Ca₁₀(PO₄)₆(OH)₂ and a primary mineral component of human bone and teeth, promoting osteoblast adhesion and bone integration^6,7,15–18^. It is used for dental tissue reconstitution due to its excellent biocompatibility, bioactivity, great osseointegration, and high osteoinductivity^6,15,19,20^. HAp has been used to coat the surfaces of Mg alloys^21,22^ biomedical austenitic stainless-steel grades, and biomedical Ti-based alloys^12,23,24^. It has also significantly enhanced the surface and corrosion properties of implants. In such scenarios, HAp maintains the mechanical properties of the substrate metal while promoting rapid osseointegration with surrounding tissues and bones^6,7,15,16,18^.

Hydroxyapatite (HA), as a primary mineral component of bone, has been extensively studied for its biocompatibility and role in improving the biological performance of implant surfaces. Its application in orthopaedics and dentistry is well-documented, especially for enhancing osseointegration and surface bonding strength with host tissues^13,25^. In addition to these advantages, recent comprehensive reviews highlighted the evolving role of HA in surface engineering of biomaterials for clinical use, emphasizing novel synthesis routes and integration strategies^26^.

In dental implants, the osteoconductivity of biomaterials is critical to bone regeneration as it affects osseointegration and prosthesis longevity. Osteoconductivity requires a functional and structural linkage between the bone and implant material without any soft tissues. The differentiation and adhesion of osteoblast during the onset of implant and later bone remodeling is largely dependent on the surface of the implant material^27,28^. While inertness is advantageous for the prevention of any negative tissue response, biointegration has led to the use of CaP coating on metallic implants^29,30^. The mechanical property of HA is size (scale) dependent^31^. Although it is brittle, some plastic response is possible at the nanoscale^32^. The use of nano biomaterials has the potential of achieving better biointegration properties and improved tribological properties. Implant longevity depends not only on osseointegration but also on the implant’s ability to withstand mechanical stresses and wear over time^33^. Thus, assessing the wear resistance after osseointegration is key for the longevity and structural integrity of the implant^34^.

Several methods, which include surface roughening and coating of Ti alloys have been used to improve the success and longevity of implants^12,23,24^. Ceramic and metallic coatings tend to create interfaces that may limit the ability of the implant to withstand masticatory forces^18,35,36^. Bioactive coatings can eliminate this by maintaining a bond with metallic implants (on the inner interface of the coating) while creating a chemical fusion between the surrounding bone on its exterior surface^37^.

Previous studies have demonstrated the potential of HA coatings in improving osseointegration^6,7,15–18,38,39^. However, the tribological properties of HAp-coated biomedical grade Ti alloys have not been adequately studied. Most existing research focuses on HA coatings’ bioactivity and adhesion, with limited attention to their wear mechanisms under physiological loading conditions. A comprehensive understanding of friction and wear mechanisms in HA-coated Ti-6Al-4V is essential for optimizing coating performance and enhancing implant durability.

This study systematically evaluates the friction and wear behavior of HA-coated Ti-6Al-4V using a novel pack cementation process. The resulting coatings are shown to enhance both osseointegration and wear resistance. This approach not only enhances coating robustness but also provides a deeper understanding of tribological performance under physiological loading. The proposed method produces a mechanically durable HA layer with an improved surface topography that may contribute to superior wear resistance. Through comprehensive material characterization, using scanning electron microscopy (SEM), energy-dispersive X-ray spectroscopy (EDS), Raman spectroscopy, and X-ray diffraction (XRD), we evaluate the structural and compositional integrity of the coatings. Additionally, nanoindentation and pin-on-disk wear testing provide insights into the friction and wear mechanisms at the implant interface. In vitro bioactivity testing in simulated body fluid (SBF) further confirms the bone-bonding capability of the coated substrates.

This research bridges a critical gap in understanding the tribological behavior of HA-coated Ti-6Al-4V, providing fundamental insights into friction and wear mechanisms, which are critical for improving the durability and biocompatibility of dental implants.

Although hydroxyapatite (HA) coatings on Ti-6Al-4V have been widely studied, prior research primarily focuses on osseointegration and coating adhesion, often overlooking tribological performance under physiological conditions. Most existing studies rely on commercially available or synthetic HA applied through conventional techniques such as plasma spraying, sol-gel processing, or electrochemical deposition^40–48^. While effective in certain aspects, these methods frequently encounter challenges related to coating durability, biocompatibility, and wear resistance^11^. To address these limitations, the present study introduces a novel, low-cost pack cementation approach using HA synthesized from sustainable biowaste sources such as bovine bone and eggshells^11^. The technique yields nanostructured coatings with enhanced morphological and chemical properties, resulting in improved tribological behavior and bioactivity. Unlike prior investigations, our study uniquely combines a sustainable biomimetic HA source with a novel deposition process and a comprehensive evaluation of tribological behavior, thereby addressing a critical knowledge gap and offering new insights for dental implant surface engineering.

Table 1 presents a comparative overview of key prior studies, highlighting how the current work advances the field through its unique combination of a green HA source, scalable coating method, and comprehensive tribological and bioactivity assessment^40–48^. By bridging this critical gap, our study offers new insights for enhancing the performance and longevity of dental implant surfaces.

**Table 1:** Comparison of previous studies on hydroxyapatite (HA) coatings on Ti-6Al-4V alloys, highlighting differences in HA source, deposition methods, and focus on tribological performance, with emphasis on how the present study addresses existing gaps.

HA sources	Deposition methods	Key findings
Supersaturated solutions of calcium and phosphate ions	Metastable precursor solutions	Controlled deposition with desirable thickness, morphology, and chemical composition at low temperatures and cost^43^
Commercial HA powder	Atmospheric plasma spray (APS) and CoBlast (nonthermal)	CoBlast showed higher tensile adhesion and better cell proliferation compared to APS^48^
Commercial HA powder	Plasma spray and CoBlast	CoBlast coatings underwent a two-step recrystallization process in SBF, while plasma spray coatings showed heterogeneous nucleation^45^
Synthesized HA powder	Detonation spraying	Different secondary phases formed depending on substrate material (PEEK vs. Ti)^49^
Commercial HA powder	Various techniques including ablation	Higher substrate temperatures improved crystallinity but degraded bioactivity above 700 °C^41^
Simulated body fluid (SBF)	Biomimetic growth	Stable HA coating formed after 13 h without SBF replenishment, biocompatible with osteoblast cells^47^
Bovine bone-derived HA	Spray pyrolysis	Superior crystallinity and nanostructured features, effective for controlled drug release^42^
Mixture of calcium fluoride and HA powders	Plasma spraying	9 wt% calcium fluoride improved mechanical properties and corrosion resistance^44^
F-and La-co-substituted HA	Anodization, electrochemical deposition, hydrothermal treatment	Enhanced chemical stability, mechanical properties, and biocompatibility with strontium titanate nanotubes reinforcement^46^

## Materials and methods

### Materials

Bovine bone and eggshell, abundant biowaste, and accessible materials were obtained from a local market in Accra, Ghana. The substrates for the study, Ti-6Al-4V plates of 2 mm thickness were procured from McMaster-Carr (Los Angeles, California, USA). The simulated body fluid (SBF) was sourced from Thermo Fisher Scientific (Waltham, MA, USA).

### Fabrication of substrates

The 2 mm thickness Ti-6Al-4V was cut into 10 mm $\:\times\:\:10\:\text{m}\text{m}\:$ substrates and polished with emery paper (from 400 grit to 1200 grit). The untreated substrates were ultrasonically washed using distilled water, ethanol, and acetone for 10 min, and then air dried. Four categories of substrates were fabricated for this study: as-received (AsR_Ti-6Al-4V), annealed Ti-6Al-4V (A_ Ti-6Al-4V), HA-coated substrates from bovine source (BHA_ Ti-6Al-4V), and HA-coated substrates from eggshell source (EHA_Ti-6Al-4V). For the A_Ti-6Al-4V samples, the cut-prepared substrates were placed in a furnace at a 10 °C/min heating ramp in an RHF 1600 Carbolite furnace (Carbolite Gero, Derbyshire, United Kingdom). The substrates underwent isothermal annealing at 900 °C for 2 h, after which the furnace was cooled to room temperature.

#### Coating preparation

##### Bovine hydroxyapatite (BHA) coated Ti-6Al-4V substrate

The BHA_Ti-6Al-4V coated substrate was prepared as follows. Bovine bones were first thoroughly washed to remove impurities and visible fat. The cleaned bones were air dried and crushed into smaller fragments. These fragments were pulverized to a powder of ~ 44 μm particle size. In a ratio of 3:1 by weight, the bovine bone powder and barium carbonate (BaCO_3_) (serving as an activation compound) were mixed. The prepared Ti-6Al-4V was placed in the mixture in an alumina crucible and treated in a furnace for 2 h at 900 °C at a rate of 10 °C/min. After the treatment, the furnace was allowed to cool naturally to room temperature.

##### Eggshell hydroxyapatite (EHA) coated Ti-6Al-4V substrate

The HA-coated substrate from eggshell (EHA_Ti-6Al-4V) was prepared by first synthesizing CaP from the eggshell. The eggshells were washed thoroughly and dried. The dried eggshells were then pulverized and calcined at 900 °C in the furnace at a rate of 10 °C/min to get calcium oxide (CaO). The resulting CaO was dissolved in deionized water and stirred at 400 rpm for 1 h. Dilute 85% phosphoric acid (H_3_PO_4_) was added to this solution to initiate precipitation. The reaction mixture was covered and left to stand for 12 h to allow complete reaction and settling. After decantation the resulting precipitate (residue) was rinsed repeatedly and dried in the oven for 12 h at 70 °C. The dried sample was crushed and pulverized to ~ 44 μm. The chemical process for the synthesis is summarized in Eqs. 1 and 2. The synthesized CaP powder and BaCO_3_ were mixed in a ratio of three to one to form a packed mixture. The pack cementation process followed the same procedure as that used for the bovine bone-based mixture.

1 $${\rm CaCO_{3(s)} Heat \rightarrow CaO_{(s)} + CO2_{(g)}}$$

2 $${\rm 3Ca(OH)_{2(s)} +2H_{3}PO_{4(aq)} \rightarrow Ca_{3}(PO4)_{2(s)} + 6H_{2}O_{(l)}}$$

## Powder and substrate microstructural characterization

The microstructure of the powders and samples was investigated under SEM (JOEL JSM – 7000 F, Golden, Co, USA). The observations were done with an accelerating voltage of 10 kV. The SEM was instrumented with energy dispersive x-ray spectroscopy (EDS) and Electron Backscatter Diffraction (EBSD) systems. The samples were gold/palladium coated by sputtering using a 2-stage vacuum pump at 230 V, 50 HZ. The EDS was used for evaluating the elemental compositions of the substrates. The size distribution of the powders and nanorods on the substrates were determined from the SEM micrographs with the ImageJ software, by outlining individual particles. The size distribution of the particles was then statistically determined.

The powders and coated substrates were analyzed for their phases with an X-ray diffraction (XRD; Empyrean, PANalytical) at 40 mA and 45 kV with 2θ angles from 20° to 80° at a counting time 2 s/step (step size of 0.002°). The International Center for Diffraction Data (ICDD) was used to determine the diffraction peaks using the High Score Plus software, Version 5.1 (PANAlytical, www.malvernpanalytical.com/en/products/category/software, Malvern PANalytical B.V., Netherlands).

The powders and substrates were successively analyzed with a high-resolution digital spectrometer, Horiba XploRa Raman Micro Spectrometer (Kisshoin, Minami-Ku Kyoto, Japan). The setup had an 1800–line grating with a 100 aperture and 300 slit width. A laser operating at 532 nm wavelength and approximately 10 mW optical power was employed for the tests. The laser light was focused using a 100x lens on an Olympus microscope integrated with the spectrometer. The calibration utilized a silicon wafer (100) with a peak of 520 cm^−1^. LabSpec 6 software Version 6.5 from Horiba (www.horiba.com/int/scientific/products/detail/action/show/Product/labspec-6-spectroscopy-suite-software-1843/, Kisshoin, Minami-Ku Kyoto, Japan) was used for normalization and baseline correction.

The topography of the substrates was analyzed using atomic force microscopy (AFM), capturing detailed images at both the micro- and nanoscale. The Park Systems NX20 (Suwon, Korea) setup was used with non-contact PPP-NCHR 10 M cantilevers having silicon nitride tips. The tip of 10 nm nominal radius and 10–15 μm length was brought very close to the substrate in a non-contact mode. The images were captured and recorded with a scanning rate of 0.17 Hz, and 1024 × 1024 pixels resolution.

### Nano-mechanical characterization

With a Berkovich tip, nanoindentation hardness (H) was measured using iMicro Triboscope nanoindentor (Milpitas, California, USA). To avoid the effects of the substrate layer on the coated layer, the depth of indentation was limited to no deeper than 1/10 of the coating layer. With a coating thickness of ~ 5 μm, the limiting penetration depth was ~ 0.5 μm. The indentation was performed at a depth of 40 nm which is well below the limiting value, and a displacement rate of 0.2 m/s. The Advanced Dynamic Hardness to Depth mode was used to measure the hardness after 10 s hold time and unloading.

### Friction and wear of substrates

Wear experiments were performed using a calibrated Anton-Paar TRB^3^ tribometer (Peseux, Switzerland). The experiments were conducted with a ball-on-disk configuration at 0.40 cm/s with a 2 N load. This load was chosen to simulate moderate physiological forces during early osseointegration, where excessive loading may not reflect in vivo conditions and could cause coating damage or mask subtle wear mechanisms. The wear rates and coefficient of friction of the AsR_Ti-6Al-4V, A_Ti-6Al-4V, BHA_Ti-6Al-4V, and EHA_Ti-6Al-4V were determined using a 6 mm 100 Cr steel balls as a counter body. The humidity of the room was 51% and the temperature of the room was ~ 23 °C. Data was collected at a frequency of 20 Hz. The experiments were performed at various time steps and the investigations of the wear tracks were done using SEM equipped with EDS. The substrate’s wear tracks were also analyzed with Raman spectroscopy to determine tribocatalysis products in them. The depth characterization of the wear tracks was done with a Taylor Hobson Surtronic S128 Profilometer (Leicester, UK). The rate of the wear of the substrates was determined based on the volume of the worn-out substrates, *V*_*w*_ which was determined from Eq. 3. The rate of wear k_x_, was determined with Eq. 4. The results of the wear rate are summarized in Table 2.

3 $$\:{V}_{w}={A}_{w}P{d}_{max}$$

4 $$\:{k}_{x}=\:\frac{{V}_{w}}{F\times\:s}$$

Where A_w_ = surface area of the worn substrate, s = sliding distance, F = load applied and Pd_max_ = average penetration depth.

### Bioactivity test of substrate

The substrates were assessed in vitro for bioactivity through immersion in the SBF. Through this evaluation, the bone-bonding capability of the substrates was ascertained. The substrates were submerged in the SBF and placed in an incubator at 37 °C and 5% C for 28 days. The SBF was replaced daily for all samples at 24-hour intervals. From the 3rd to the 7 th day, the SBF was changed every 48 h. Thereafter, the SBF was replaced every 72 h until day twenty-eight. The samples were removed from the solution after the soaking period, gently washed with distilled water, and air-dried at room temperature. To qualitatively assess bioactivity, the substrates’ surface morphology and elemental composition were examined using SEM/EDS. While SEM provided insight into morphological changes resulting from SBF interaction, the EDS elemental maps confirmed the presence of calcium and phosphorus within the surface deposits. The combined SEM/EDS approach was used to substantiate the formation of bone-like apatite and interpret the bioactive response of the coatings.

## Results and discussion

### Microstructural characterization of powders

**Fig. 1:**
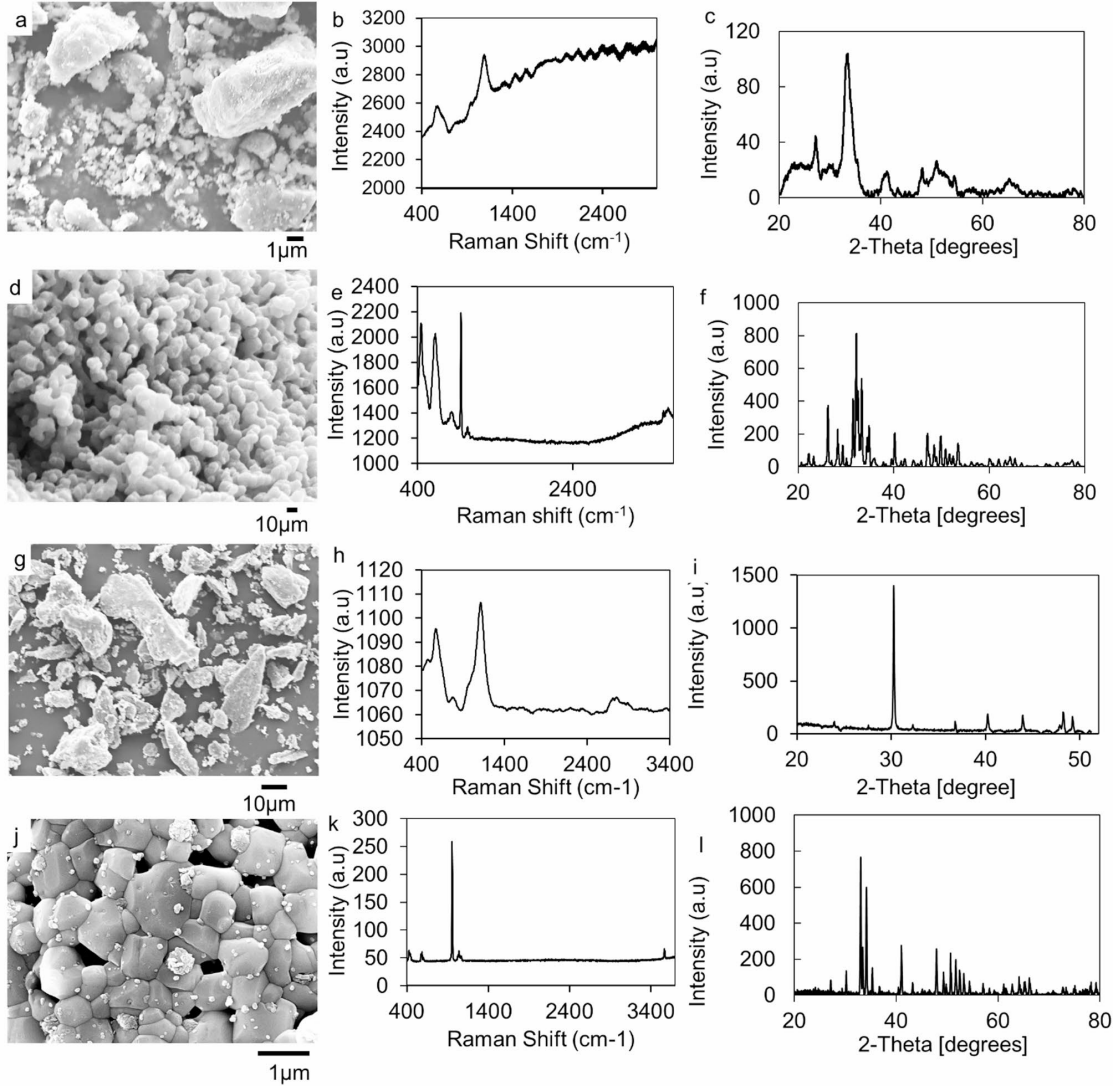
Micro- and structural analyses of bovine bone, BHA, eggshell powder, and EHA: (**a**) SEM of eggshell, (**b**) Raman spectrum of eggshell, (**c**) XRD diffractogram of eggshell; (**d**) SEM of EHA, (**e**) Raman spectrum of EHA, (**f**) XRD diffractogram of EHA; (**g**) SEM of bovine bone, (**h**) Raman spectrum of bovine bone, (**i**) XRD diffractogram of bovine bone; (**j**)SEM of BHA, (**k**) Raman spectrum of BHA, (**l**) XRD diffractogram of BHA.

The morphology of the eggshell powder as shown in Fig. 1a exhibits irregularly shaped particles. Figure 1b showed a broad Raman absorbance band at 1091 cm^−1^ indicating the v1 mode of calcium carbonate^50^. The eggshell powder’s crystal structure and chemical composition were ascertained by XRD analysis. The XRD pattern showed the calcite (CaCO_3_) phase for the eggshell powder in Fig. 1c. The sharpest diffraction peaks appeared at 2$\:\theta\:={30.27}^{o}$ with plane (202). The other peaks with their respective planes are: 36.80$\:^\circ\:$, 40.24$\:^\circ\:$, 43.96$\:^\circ\:$, 48.26$\:^\circ\:$, and (310), (013), (111), (112). This was in good agreement with CaCO_3_ (ICDD card number 51–1524).

Figure 1d shows the microstructure of EHA with rod-like particles with a particle size distribution of 67.11 $\:\pm\:$12.11 nm. Raman spectra for EHA in Fig. 1e revealed peaks at 446 cm^−1^, 633 cm^−1^, 840 cm^−1^, 957 cm^−1^, 1069 cm^−1^, and 3648 cm^−1^. The strongest band at 957 cm^−1^ is attributed to the symmetric stretching ν1 mode of$\:\:{\text{P}\text{O}}_{4}^{3-}$ band. The peaks at 446 cm^−1^ and 1069 cm^−1^ correspond to ν2 bending and ν3 antisymmetric stretching modes, respectively, of $\:{\text{P}\text{O}}_{4}^{3-}$ion. The 840 cm^−1^ and 1069 cm^−1^ also indicate the stretching mode of the B-type carbonate group. The low-intensity peak detected at 3648 cm^−1^ indicates the existence of OH^−^ ions within HA. The nature of the chemical bond in the materials as described by the Raman spectra confirms the synthesis of B-type carbonated HA^51^. Figure 1f shows the pattern of XRD for EHA. The phase analysis was conducted using ICDD standard HA with PDF card number 086–0740 for the hexagonal HA structure. The prominent diffraction peaks at 2θ values 22.3, 26.2, 28.3, 29.3, 32.1, 33.3, 34.8, 40.2, 48.4, 49.8, 50.8, 52.3, corresponding to the (111), (002), (102), (120), (112), (300), (202), (130), (132), (213), (231), (402) Millar planes. The results closely align with HA (ICDD card number 9–0432)^52,53^.

From Fig. 1g the SEM micrograph of bovine bone showed irregular particles. The Raman spectra for the bovine bone sample in Fig. 1h showed bands at 579 cm^−1^, 785 cm^−1^, 1090 cm^−1^, and 2441 cm^−1^. The existence of phosphates in the bovine bone is distinguished by ν4 bending mode at 579 cm^−1^, and ν3 antisymmetric stretching mode at 1090 cm^−1^. The bovine bone’s XRD pattern is shown in Fig. 1i. A broad diffraction peak with the highest intensity is seen at 2θ values with 33.3 cm^−1^. There were other peaks at 41.2 cm^−1^ and 52.3 cm^−1^. The amorphous nature of the bovine bone from its organic components (collagen) was depicted by the broad peak^54^.

From Fig. 1j the SEM image of calcined bovine bones at 900^o^C showed hexagonal particles with a particle size of 0.541 $\:\pm\:$ 0.150 μm. Raman spectra for the BHA in Fig. 1k showed peaks at 442 cm^−1^, 583 cm^−1^, 957 cm^−1^, 1040 cm^−1^, and 3570 cm^−1^. The strongest band at 957 cm^−1^ also corresponds to the symmetric stretching ν1 mode of$\:\:{\text{P}\text{O}}_{4}^{3-}$ band. The peaks at 442 cm^−1^ and 1040 cm^−1^ corresponds to ν2 bending mode and ν3 antisymmetric stretching mode of $\:{\text{P}\text{O}}_{4}^{3-}$ band. The peak at 1040 cm^−1^ is also an indication of the B–type carbonate apatite, which is synonymous with the carbonate apatite signature of biomaterial from young bone tissue^55^. The XRD spectrum of the BHA from Fig. 1l had characteristic peaks at 2θ near 27.2^o^, 30.1^o^, 33.0^o^, 34.2^o^, 35.3^o^, 36.7^o^ corresponding to the (102), (211), (300), (202), (301) Millar planes. This was in good agreement with HA (ICDD card number 9–0432)^52,53^.

### Microstructural characterization of substrates

**Fig. 2:**
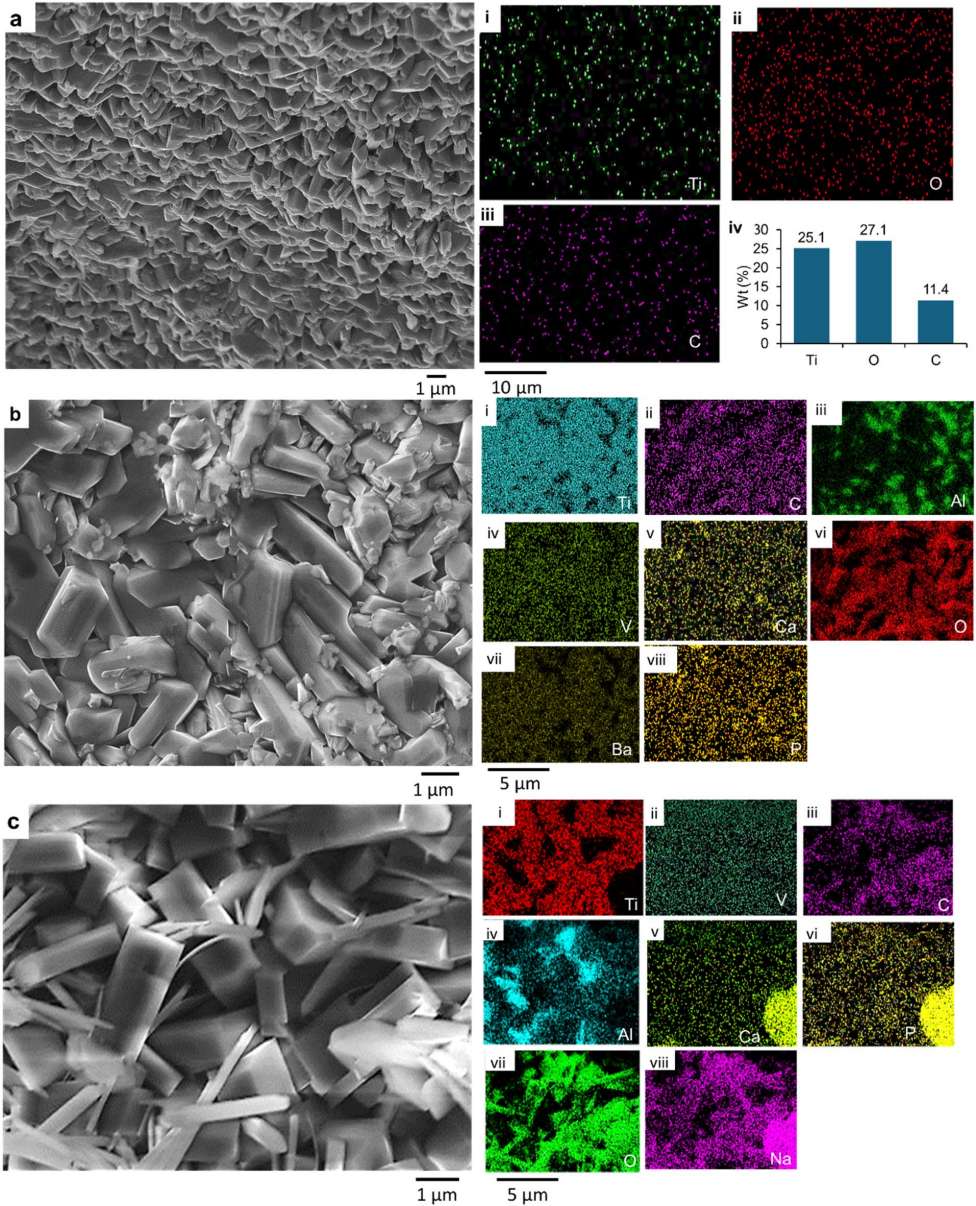
SEM images of annealed and coated substrates. (**a**) A_Ti-6Al-4V with EDS spectra (i – iii); (**b**) EHA_Ti-6Al-4V with EDS spectra (i – viii), (**c**), BHA_Ti-6Al-4V with EDS spectra (i – viii).

**Fig. 3:**
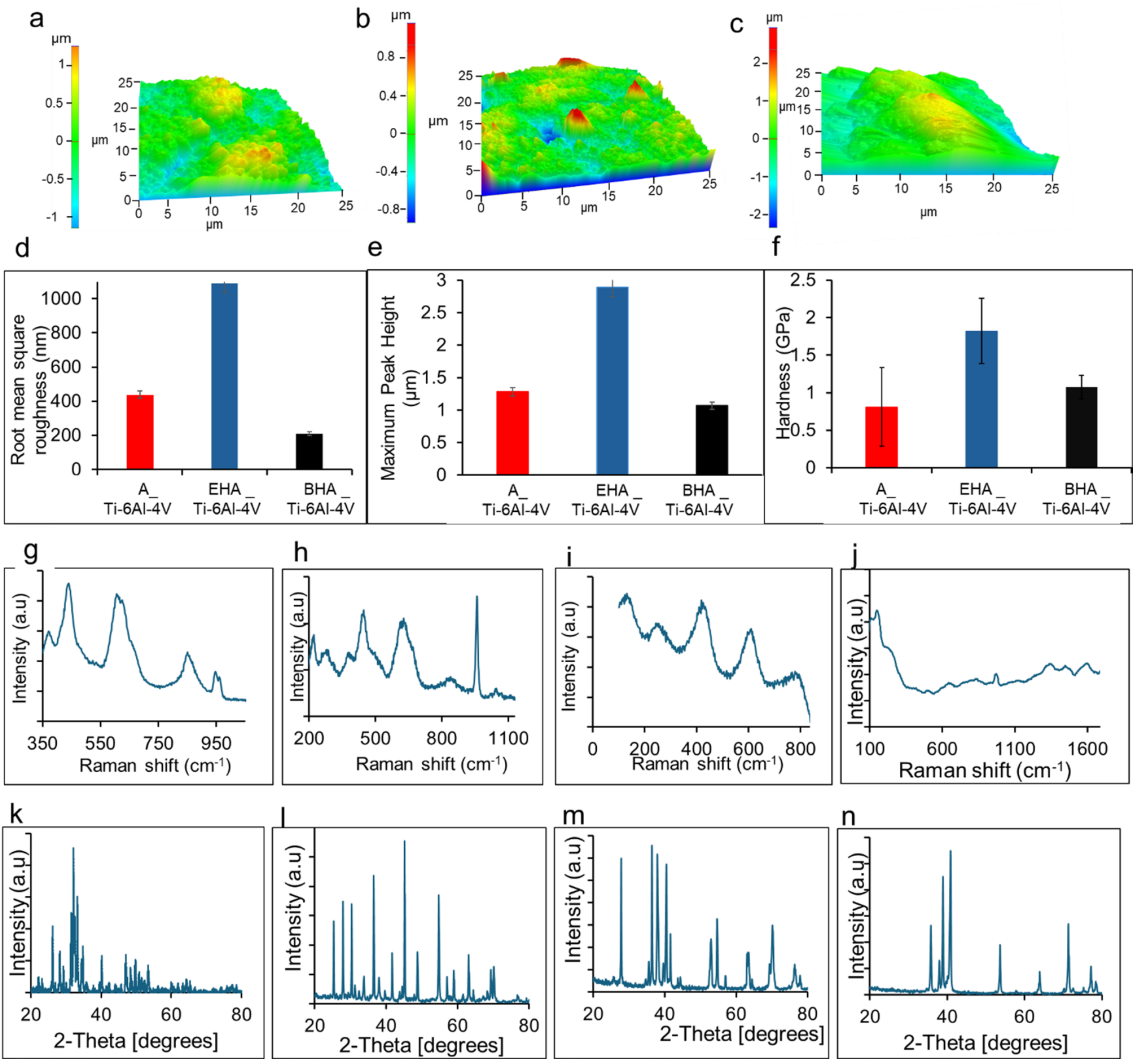
Non-contact mode AFM showing (**a**) A_Ti-6Al-4V; (**b**) EHA_Ti-6Al-4V, (**c**) BHA_Ti-6Al-4V. (**d**) RMS roughness for the substrates, (**e**) maximum deposited height of coated substrates, (**f**) hardness results for substrates; Raman spectra of treated substrates (**g**) EHA_Ti-6Al-4V, (**h**) BHA_Ti-6Al-4V, (**i**) A_Ti-6Al-4V, (**j**) AsR_Ti-6Al-4V. XRD diffractogram of treated substrates (**k**) EHA_Ti-6Al-4V, (**l**) BHA_Ti-6Al-4V, (**m**) A_Ti-6Al-4V, (**n**) AsR_Ti-6Al-4V.

The morphology of the nanostructures on the A_Ti-6Al-4V substrate had quasi-spheroidal microparticles as shown in Fig. 2a. The AFM image of this substrate as shown in Fig. 3a revealed irregular surfaces with a root mean square value of 438.2 nm. The maximum height of the nanorods on the substrate was 1286 nm. The chemical composition of the samples analyzed via SEM/EDS as shown in Fig. 2a(i - iii) revealed the existence of O and Ti. The Raman spectra of the 900 °C annealed substrate A_Ti-6Al-4V in Fig. 3i had peaks at 260 cm^−1^, 430 cm^−1^, 603 cm^−1,^ and 786 cm^−1^, indicating the presence of rutile phase of TiO_2_ on the substrate after the annealing process. The XRD pattern of the substrate in Fig. 3m showed characteristic peaks at 27.8° 36.5°, 38.1°, 40.5°, 41.6°, 53.1°, 54.7°, 63.7°, 70.5°, 76.7°, 78.2° corresponding to (001), (002), (011), (102), (110), (103), (112), (201). One higher peak intensity was matched to a titanium oxide of the plane (001) at 27.8° which corresponded to ICDD: 04–007-4073. The remaining higher peak intensities planes (100), (002), (011), (102), (103) at 36.5°, 38.1°, 40.5°, 54.7°, 70.5°, corresponded to titanium oxide ICDD: 04–015-8643. The nanoindentation hardness of the substrate in Fig. 3f was 0.811 GPA.

The coating’s morphology on the EHA_Ti-6Al-4V substrates had rod-like nanoparticles as shown in Fig. 2b. The surface roughness examined by the AFM image of EHA_Ti-6Al-4V as shown in Fig. 3b revealed irregular rough surfaces, with a root mean square value of 208.95 nm. The maximum height of the nanorods on these substrates was 1069 nm. The chemical composition of the coatings analyzed via SEM/EDS as shown in Fig. 2b(i-viii) revealed the presence of substrate elements like Ti, Al, and O and coating elements such as P and Ca. The presence of P and Ca indicates the successful coating of HA on the Ti-6Al-4V metal. The Raman spectra of the EHA_Ti-6Al-4V substrates as shown in Fig. 3g had bands at 447 cm^−1^, 612 cm^−1^, 962 cm^−1^, 3880 cm^−1^, and 3645 cm^−1^. The bands at 447 cm^−1^ and 612 cm^−1^ are characteristics of the rutile phase. The band at 962 cm^−1^ is attributed to the symmetric stretching ν1 mode of$\:\:{\text{P}\text{O}}_{4}^{3-}$ band. The peak at 3645 cm^−1^ also shows the presence of OH^−^ ions in the HA. The XRD pattern of the substrate in Fig. 3k showed characteristic peaks at 25.4°, 35.5°, 38.2°, 41°, 43°, 44°, 52°, corresponding to (201), (300), (220), (212), (113), (400). This matched the ICDD file number 01–086-0740 for the hexagonal crystal system of HA. The nanoindentation hardness of these substrates in Fig. 3f was 1.820 GPa.

The SEM micrograph of the coating on the BHA_Ti-6Al-4V substrates revealed rod-like nanoparticles, as shown in Fig. 2c. The AFM image of the BHA_Ti-6Al-4V substrates as shown in Fig. 3c reveals irregular rough surfaces of a root mean square value of 1090 nm (Fig. 3d). The maximum height of the nanorods on these substrates was 2896 nm (Fig. 3e). The composition of the coated substrates was analyzed via SEM/EDS, as shown in Fig. 2c(i – viii), revealing the existence of Ti, Al, O, Ca, and P from the substrate and coating material. The existence of Ca and P indicates the successful coating of HA on the substrates. The detected sodium (Na) is a trace element in the coating material. The Raman spectrum of the coatings on the BHA_Ti-6Al-4V substrates as shown in Fig. 3h had peaks at 137 cm^−1^, 213 cm^−1^, 442 cm^−1^, 615 cm^−1^, 845 cm^−1^, 951 cm^−1^, 3638 cm^−1^, and 3875 cm^−1^. The peak observed at 137 cm^−1^ is characteristic of the anatase phase. Peaks at 213 cm^−1^, 442 cm^−1^, and 615 cm^−1^ are characteristics of rutile phase. The peak at 951 cm^−1^ also corresponds to the symmetric stretching ν1 mode of$\:\:{\text{P}\text{O}}_{4}^{3-}$ band, and that at 3638 cm^−1^ shows the existence of OH^−^ ions in the HA. The XRD pattern of the substrate as shown in Fig. 3l showed characteristic peaks representing Titanium oxide and HA. Titanium oxide peaks that matched 04–007-4073 were identified at 27.5, 44.5, 56.8, 68.3 corresponding to (001), (101), (002), (111). The HA peaks matched HA ICDD: 04–017-1626 at 25.5°, 31.2°, 32.3°, 33.7°, 41.7°, 45.1°, 48.9°, 55.1° corresponding to (002), (211), (300), (202), (302), (401), (213), (322). The nanoindentation hardness of the substrates, as shown in Fig. 3f was 1.075 GPa.

The Raman spectrum of the AsR_Ti-6Al-4V substrate in Fig. 3j had low peaks at 141 cm^−1^ and 231 cm^−1^, which are characteristics of anatase and rutile respectively^56^. The XRD pattern of the AsR_Ti-6Al-4V substrate in Fig. 3n showed characteristic peaks at 35.7°, 38.6°, 38.8, 40.8°, 53. 6°, 63.8°, 71.2°,77.1° (1 0 0), (0 0 2), (1 0 1), (1 0 2), (1 1 0), (1 0 3), (112). There was a 100% match with ICDD file number 04–020-7055 which is a hexagonal crystal system of Ti-6Al-4V.

### Wear and friction properties

**Fig. 4:**
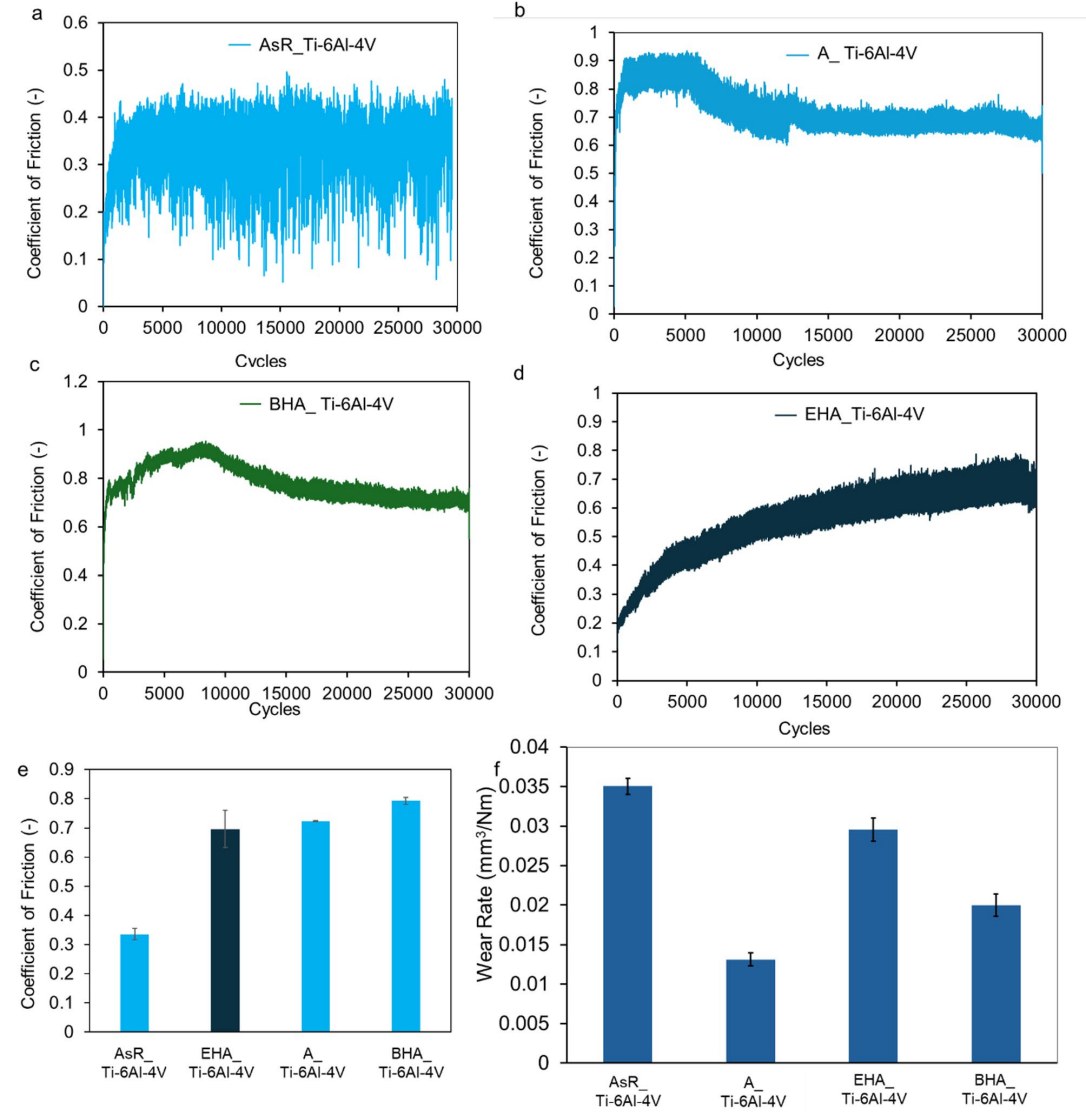
Friction coefficient versus cycles plot of, (**a**) AsR_Ti-6Al-4V, (**b**) A_Ti-6Al-4V, (**c**) BHA_Ti-6Al-4V (**d**) EHA_Ti-6Al-4V, (**e**) average coefficient of friction plot of the coated and uncoated substrates, (**f**) wear rate of substrates.

**Fig. 5:**
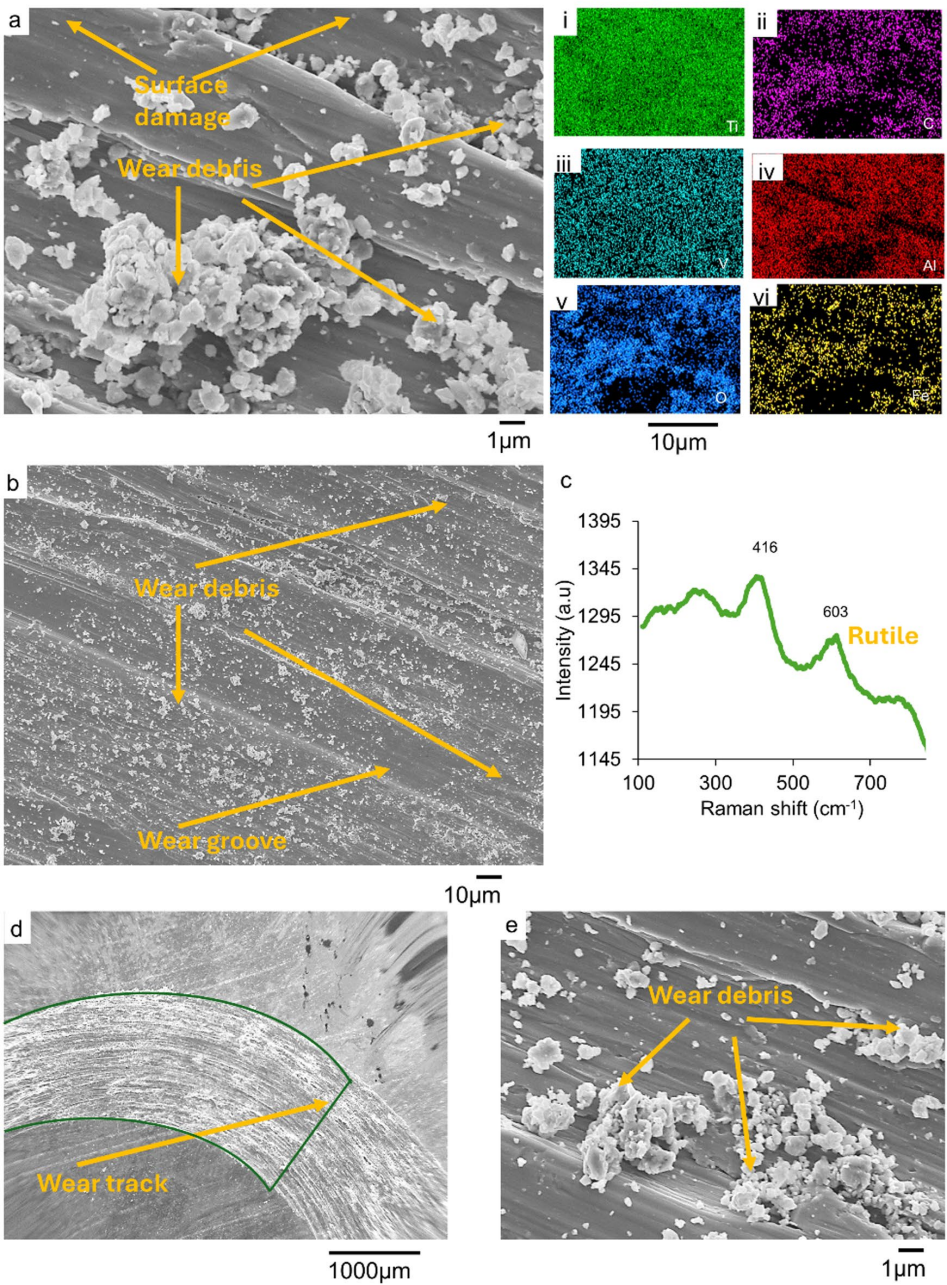
SEM images and EDS results of 30 min track of A_Ti-6Al-4V (**a**) SEM micrographs of 30 min track showing the full wear track on the substrate, (**b**) - (**c**) SEM micrographs of 30 min tracks showing the presence of TiO_2_ films with cracks and surface damage, (i – iii) EDS mapping of 30 min track of A_Ti-6Al-4V, showing O, Fe and Ti in the tracks, (**d**) EDS compositional analysis wear tracks. (**e**) Raman spectrum of A_Ti-6Al-4V wear track.

**Fig. 6:**
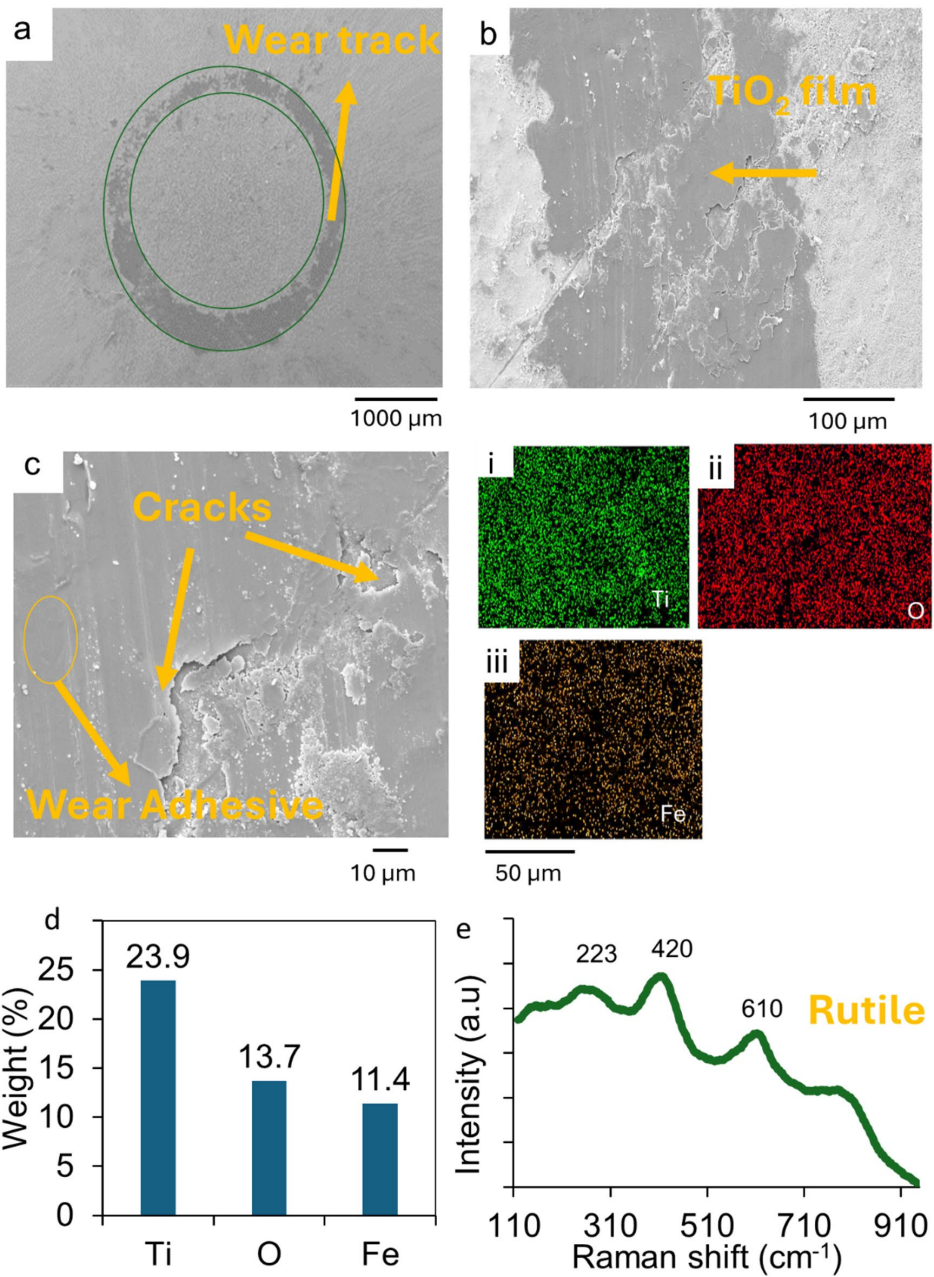
SEM micrograph of 30 min track of BHA_Ti-6Al-4V at (**a**) high magnification, (i – iv) EDS 30 min track of BHA_Ti-6Al-4V, showing Al, Fe, O, and Ti in the tracks (**b**) low magnification (**c**) Raman spectrum of BHA_Ti-6Al-4V wear track.

**Fig. 7:**
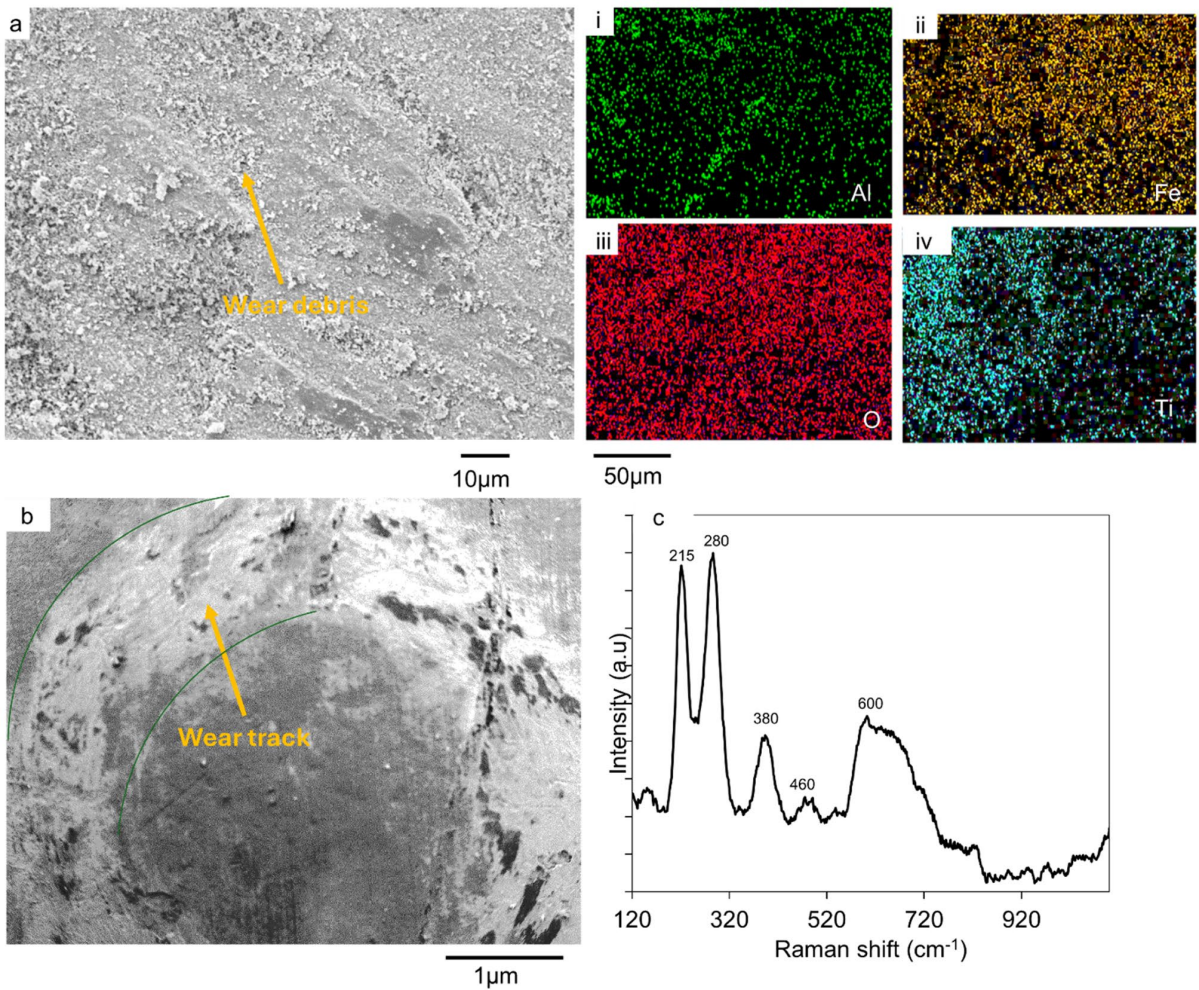
SEM micrograph of (**a**) 30 min track of EHA_Ti-6Al-4V with (i - viii) EDS mapping 30 min wear track of EHA_Ti-6Al-4V, showing the existence of P, Ti, O, Ca, Fe, C in the tracks. (**b**-**c**) SEM images of 17 min tracks of EHA_Ti-6Al-4V substrate, (**d**) Raman spectrum of 30 min tracks of EHA_Ti-6Al-4V substrate.

The coefficient of friction (CoF) versus the number of cycle plots of the substrates under 2 N load are shown in Fig. 4. The CoF curve of the AsR_Ti-6Al-4V (Fig. 4a) plunged at the beginning of the friction test before stabilizing at a CoF value of 0.35 after ~ 1300 cycles. This CoF value was then maintained for ~ 30,000 cycles. Figure 4b is the CoF curve for the A_Ti-6Al-4V substrate, which shows an initial plunging regime up to ~ 880 cycles. The curve then stabilized at a CoF value of 0.84 for ~ 6000 cycles before gradually dropping to a CoF value of 0.67. The CoF value was then constant for the rest of the test. The CoF curve for the BHA_Ti-6Al-4V substrates as shown in Fig. 4c plunged and peaked at a value of 0.92 after 8463 cycles. The CoF value then dropped gradually to 0.73 after 15,454 cycles. The CoF curve for the EHA_Ti-6Al-4V in Fig. 4d showed an initial steep rise to a CoF value of 0.4 before reaching 4040 cycles. After which it rose steadily to a CoF value of 0.68 after 29,328 cycles. The initial plunging regimes were associated with the interaction of the counter body with the TiO_2_ and HA nanorods on the substrate. This led to the crushing and smoothing of these rodlike structures to form a film that was made up of elements from the ball, coating materials, and substrates (Figs. 5, 6 and 7). Similar mechanisms have been observed in the wear of TiO_2_ nanoparticle-coated surfaces^57,58^. The bulk chemistry of these films and the presence of the nanoparticles led to varying roughness in the wear tracks which resulted in the initial rise of the friction coefficient values for the high temperature treated substrates. The initial rise and drop of the CoF were observed in the friction results of the BHA_Ti-6Al-4V and A_Ti-6Al-4V substrates.

The average friction coefficient of the substrates as shown in Fig. 4e for AsR_Ti-6Al-4V, A_Ti-6Al-4V, EHA_Ti-6Al-4V, and BHA_Ti-6Al-4V were 0.3527 ± 0.02, 0.7232 ± 0.002, 0.6961 ± 0.064, and 0.7924 ± 0.012, respectively. This is summarized in Table 2. The CoF values of the treated substrates were higher than those of the untreated substrates. For dental implants, an increase in CoF is desirable for the early stability of the bio-implant as it tends to reduce the micromotion of implant material and bone. This initial stability helps improve osseointegration which is an advantage for the success of implants^11,59^. Without adequate osseointegration, implants are merely considered to be surviving instead of being successful.

**Table 2:** Tribological properties of substrates with coefficients of friction and wear rates.

	CoF	Wear rate
AsR_Ti-6Al-4V	0.3527 ± 0.02	0.0350
A_Ti-6Al-4V	0.7232 ± 0.002	0.0131
BHA_Ti-6Al-4V	0.6961 ± 0.064	0.0200
EHA_Ti-6Al-4V	0.7924 ± 0.012	0.0295

The formation of tribocatalytic oxide films and HA residue layers in the wear tracks indicates complex interactive wear behavior between the coating and counter body. Such mechanisms, involving both adhesive and abrasive components, are consistent with recent findings in biomedical tribology underscoring the importance of surface chemistry and nanostructure in determining wear behaviour^35,59^. These behaviours align with broader discussions on tribomechanical performance of HA-coated biomaterials, where interfacial dynamics significantly influence implant longevity and performance^60^.

### Substrates and ball wear

**Fig. 8:**
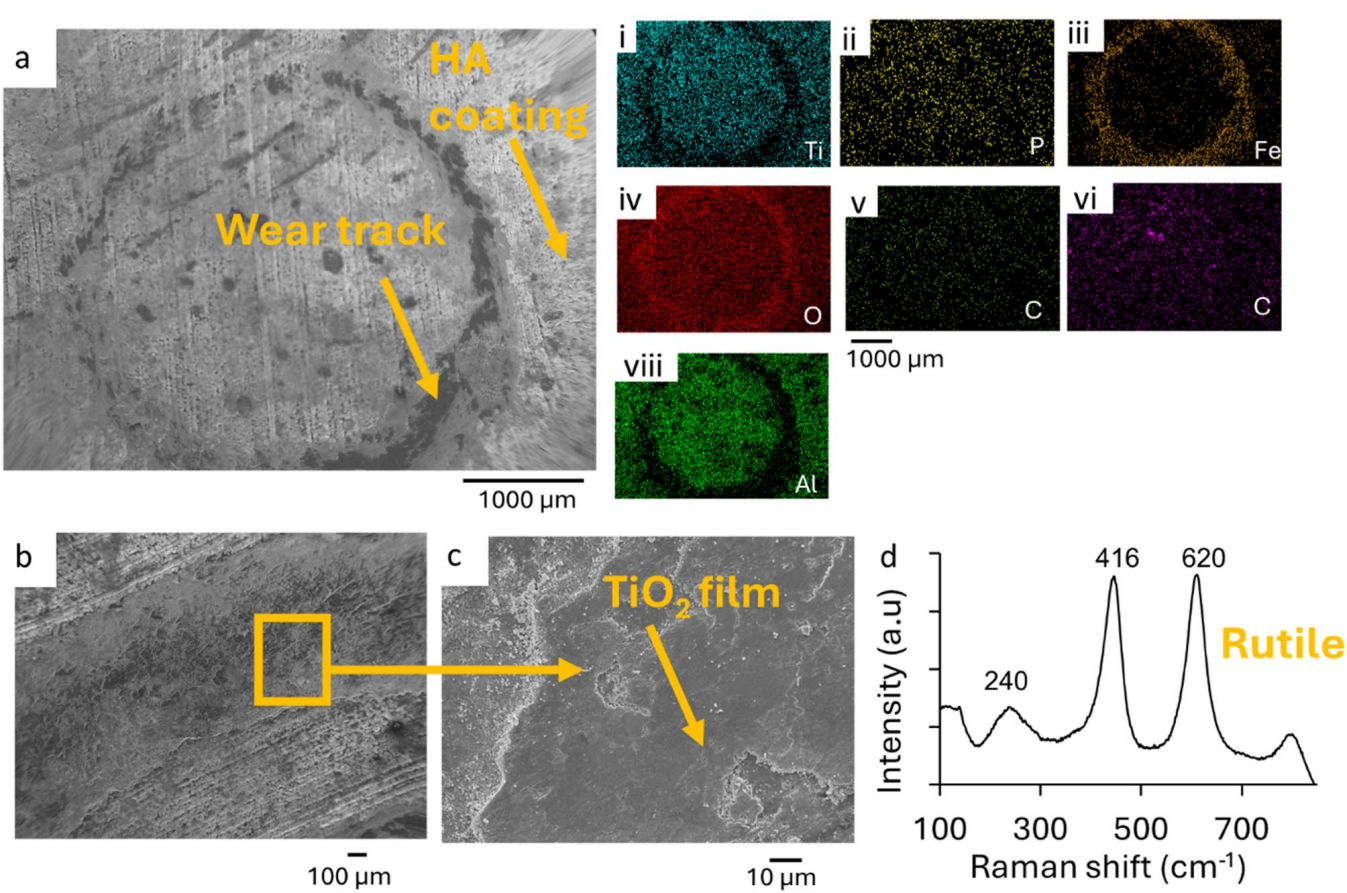
SEM micrograph of the 30 min track of AsR_Ti-6Al-4V at (**a**) higher magnification, (i - vi) with EDS spectra of AsR_Ti-6Al-4V, showing the existence of Ti, Al, C, O, V, and Fe in their respective in the tracks; (**b**) low magnification (with (**c**) Raman spectrum of AsR_Ti-6Al-4V wear track) whereas (**d**) and (**e**) are low and high magnification.

The SEM micrographs and EDS results of the wear tracks of the annealed and coated substrates as shown in Figs. 8, 5, 6 and 7 reveal the different wear mechanisms in the wear of the substrates. Generally, the interaction between the nanorods on the treated substrates led to the development of tribocatalysis products in the tracks made by the wear process. The nanorods coatings’ presence also modified the substrates’ wear rates.

Figure 8 (a, b, d, and e) are SEM images of the tracks on the AsR_Ti-6Al-4V substrates after 30 min of wear. The images reveal micro groves, material damage, microcracks, and material transfer. These are characteristics of abrasive wear mechanisms that lead to high wear rates. The images also show wear debris on the tracks. The EDS analysis of the debris as shown in Fig. 8 (a(i-vi)) showed that oxides of Fe and Ti, which were tribological oxidation products. This is evidence of the adhesive wear mechanism, which results from the interactive action of the ball and substrate materials in the presence of nanoscale surface oxide layers^11,59^. The interplay between the adhesive and abrasive wear mechanisms led to the highest wear rate in these substrates, compared to those for the treated conditions. The Raman spectrum of the track as in Fig. 8c had characteristic peaks at 416 cm^−1^, 603 cm^−1^, and 773 cm^−1^. The peaks correspond to the rutile phase of titanium oxide^57,58^.

The SEM micrograph of the track on the A_Ti-6Al-4V treated substrates, as depicted in Fig. 5 (a-c), reveals the formation of tribocatalytic films in the wear tracks. The films had cracks in them and uniformly covered the tracks on the substrates. This prevented abrasive wear on the wear tracks, thereby improving the material’s wear resistance. The EDS mapping of the film in Fig. 5(c(i-iii), d) revealed Ti, O and Fe, which was an indication of oxide-oxide interactions resulting in adhesive wear. The EDS analyses indicated the presence of Ti from the substrate element and Fe from the counter body. The Raman spectrum of the wear track as depicted in Fig. 5(e) had characteristic peaks at 223 cm^−1^, 420 cm^−1^ and 610 cm^−1^. These peaks represent the rutile phase of titanium oxide peaks.

The SEM micrograph of the 30 min track on the BHA_Ti-6Al-4V substrate is also shown in Fig. 6(a). The wear tracks on this substrate show wear debris which were distributed randomly in it. The wear tracks did not exhibit significant abrasive wear as seen in the AsR_Ti-6Al-4V substrate’s wear. The qualitative EDS results in Fig. 6(a(i-iv) showed a high concentration of Ti, Fe and O in the wear debris. This is an indication of titanium oxides and iron oxides in the wear debris. This reveals adhesive wear mechanism which is motivated by oxide-oxide interactions between the substrate and counter body. Figure 6(b) is a 60 min wear track of the substrate that shows similar interactions in tracks made by the abrasion process.

The Raman spectrum of the tracks as shown in Fig. 6(c) had characteristic bands at 215 cm^−1^, 280 cm^−1^, 380 cm^−1^, and 600 cm^−1^. These peaks represent the rutile phase of titanium oxide peaks and the iron oxide phases. These were associated with the wear debris as seen in the SEM images. The peaks confirm the presence of TiO_2_ and Fe_2_O_3_ in the wear tracks. These oxides are tribological oxidation products that form in the wear tracks as the substrate material interacts with the counter body in the presence of atmospheric oxygen.

The SEM micrograph of the 30 min wear track of the EHA_Ti-6Al-4V substrate is depicted in Fig. 7a. This SEM image and EDS results in Fig. 7a(i-viii) show a covering of the wear tracks with a tribological film which had high concentration of Fe, O, C, P and Ca and limited concentrations of Ti and Al. This indicates the development of tribological product film above the substrate material in the wear tracks. The source of the Fe and C was associated with the 100 Cr steel ball used for the ball-on-disk tests, while the O was obtained from the atmosphere. The deposited HA was the source of the P and Ca. The intercalation of the tribological film between the ball and substrate reduced the wear rate and increased the friction coefficient. The wear tracks did not show evidence of abrasive wear mechanisms at this point in the tribological tests.

Figure 7(b-c) presents micrographs of the EHA_Ti-6Al-4V substrate’s tracks, analyzed after 60 min of wear. These images show evidence of adhesion wear with debris of oxidized iron and Titanium oxide in the wear tracks. The adhesive and abrasive interactions between the mating surfaces after the tribological products were removed formed micro groves, cracks, and surface damage in the wear tracks. The average wear rates of these substrates were 0.0295 ± 0.0015 mm^3^/Nm. The EDS mapping of these wear tracks showed Ti, O, Al, and Fe. This suggests the removal of the HAp leaving behind the titanium oxide and formation of iron oxide. Raman analysis was done to ascertain this; the Raman spectrum of the wear track after 60 min had characteristic bands at 215 cm^−1^, 450 cm^−1^, 615 cm^−1^, and 781 cm^−1^. These bands indicate the presence of TiO_2_ on the substrate. The HA peaks that existed before the wear test had disappeared. This implies that the HA coat was worn off.

**Fig. 9:**
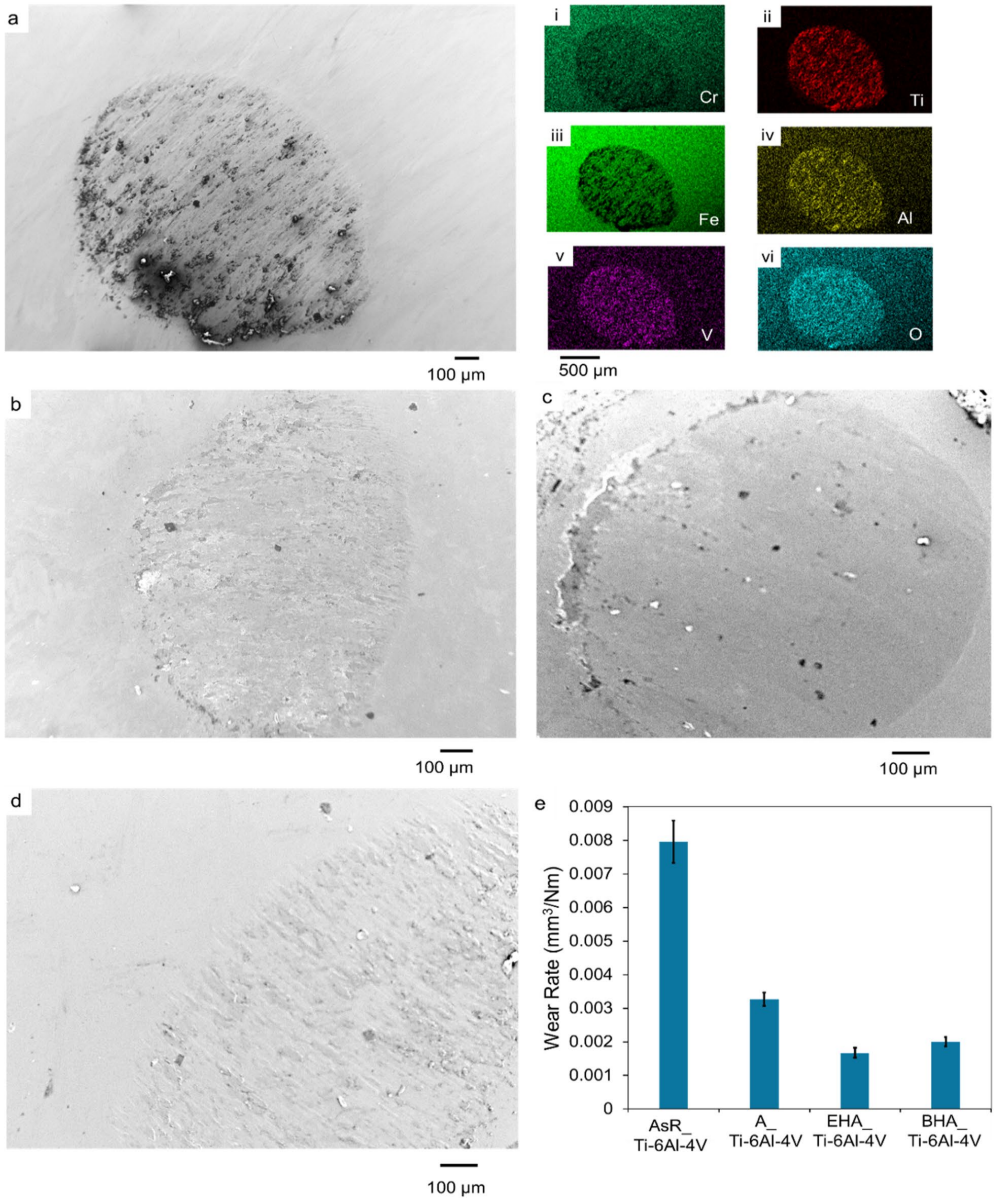
SEM images and EDS results of wear balls for the wear experiment; (**a**) SEM image of wear scar on 100 Cr steel ball used for wear of AsR_Ti-6Al-4V, (i)-(vi) EDS results for wear scar for Cr, Ti, Fe, Al, V and O respectively, (**b**) SEM micrograph of the scar on 100 Cr steel ball used for wear of EHA_Ti-6Al-4V, (**c**) SEM micrograph of scar on 100 Cr steel ball used for wear of BHA_Ti-6Al-4V (**d**) SEM micrograph of scar on 100 Cr steel ball used for wear of the A_Ti-6Al-4V substrate (**e**) wear rate of ball material for the coated and uncoated substrates.

Figure 9(a) is an SEM micrograph of the scar on the 100 Cr steel ball used to wear the as-received substrates. This micrograph reveals micro groves with randomly distributed debris in the wear scar. The EDS results in Fig. 9a(i-vi) show Ti, Al, Fe, V, O and Cr in the scar. The Ti, Al, and V are abrasive products picked up from the substrate material during the contact between the substrate and ball. The presence of the O in the tracks affirms titanium oxide and iron oxide in the substrates’ tracks.

Figure 9(b) shows a scar on the ball used for the wear of the EHA_Ti-6Al-4V substrates, this micrograph also reveals micro groves with oxide-oxide interactions. The scar on the counter body used for the BHA_Ti-6Al-4V substrate’s wear (as shown in Fig. 9(c)) reveals fewer micro groves with debris accumulation at the edges of the wear scar. Figure 9(d) is an SEM image of the wear scar of the counter body that was used for the wear of the annealed Ti-6Al-4V substrates. It reveals multiple micro groves oriented in the direction of wear. The scars of the treated balls do not show significant material damage and wear debris normally associated with abrasive wear mechanisms. This indicates a change in mechanism due to the presence of TiO_2_ and hydroxyapatite nanorods.

The coating system developed through the pack cementation method forms a distinct double-layer architecture that plays a critical role in ensuring both initial bioactivity and long-term stability. This structure comprises an inner rutile-phase TiO₂ layer thermally grown on the Ti-6Al-4V substrate during the high-temperature treatment. Additionally, there is an outer hydroxyapatite (HA) nanorod layer deposited from biowaste-derived calcium phosphate precursors. The TiO₂ layer offers strong adhesion, chemical compatibility, and corrosion resistance, while the HA layer mimics bone mineral chemistry and supports rapid apatite formation, as confirmed by SBF immersion results.

The HA layer gradually wears off, revealing the underlying TiO₂ surface upon the sliding action. This transition zone preserves surface reactivity and prevents direct metal exposure, supporting continued osseointegration. The double-layer system thus offers a synergistic advantage: bioactivity-driven early fixation from HA, followed by mechanical durability and long-term biofunctionality sustained by the TiO₂ underlayer.

The wear rate of the counter body as used against the annealed and coated substrates is presented in Fig. 9(e). The wear rate of the AsR_Ti-6Al-4V, A_Ti-6Al-4V, EHA_Ti-6Al-4V and BHA_Ti-6Al-4V were 0.008 ± 0.00064 mm^3^/Nm, 0.0033 ± 0.000196 mm^3^/Nm, 0.0017 ± 0.00015 mm^3^/Nm and 0.002 ± 0.00014 mm^3^/Nm, respectively. These indicated wear rates reduction of 58.75%, 78.75, and 75% for the balls used for the wear of the A_Ti-6Al-4V, EHA_Ti-6Al-4V and BHA_Ti-6Al-4V substrates, respectively. Therefore, the wear rates of the ball material were higher in those used for the wear of the as-received substrates than in those used on the treated substrates. The wear rate of the ball material for the annealed substrates was also higher than that for the substrates with HA nanorods. The rate of wear of the ball on the EHA_Ti-6Al-4V substrates was, however, lower compared to BHA_Ti-6Al-4V substrates.

The variations in the wear rates were also attributed to the presence of TiO_2_ and hydroxyapatite nanorods on the substrates before the wear experiment (as shown in Fig. 2). This led to the formation of tribocatalytic films (Fig. 5) in the wear tracks of the annealed substrates as observed in prior work^61^. Adhesion wear mechanisms (Figs. 5, 6 and 7) were observed on the treated substrates, and this also led to the reduction in the wear rates of the ball material^59^.

### Bioactivity of substrates

**Fig. 10:**
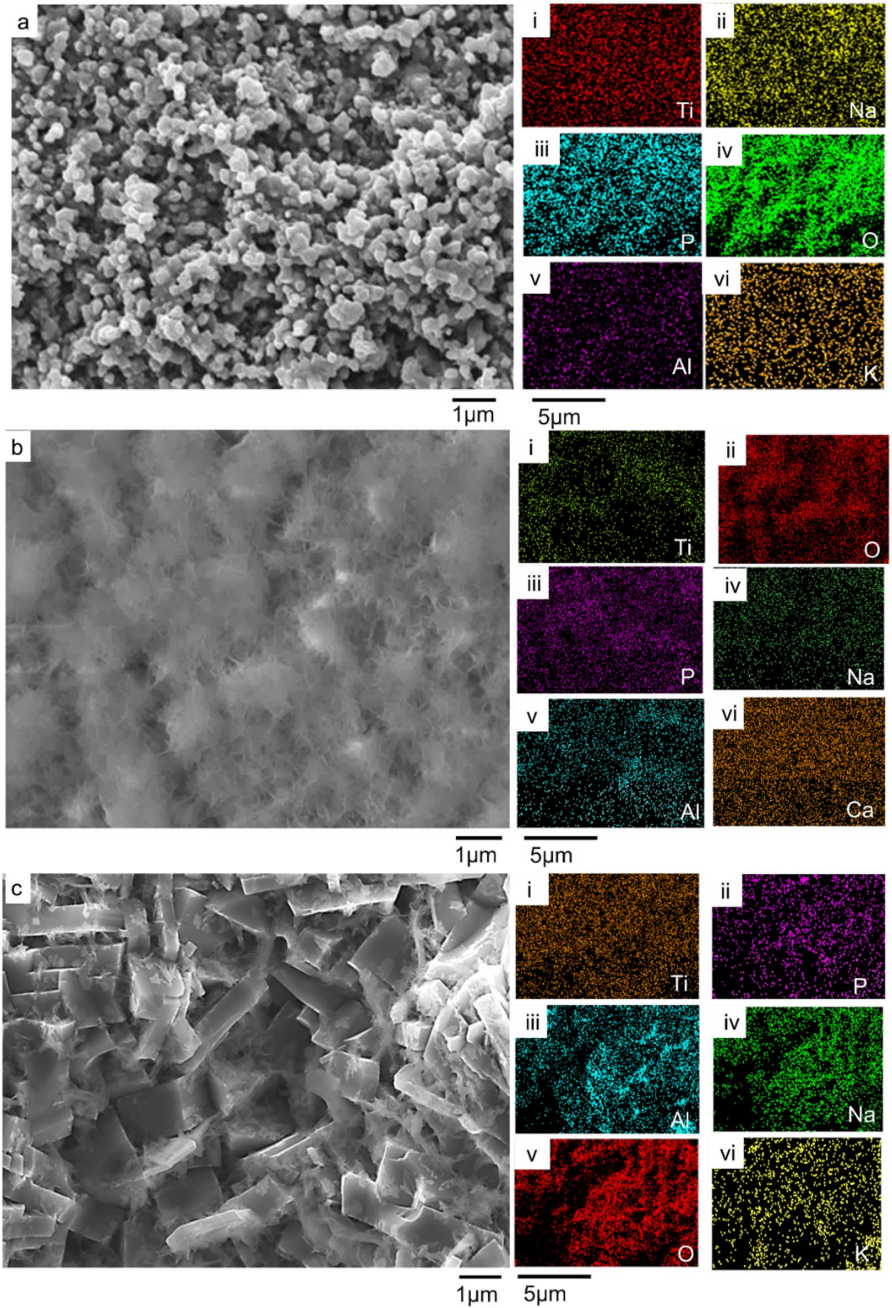
SEM micrograph of substrates submerged in SBF for 24 h of (**a**) A_Ti-6Al-4V, (i - vi) EDS results for A_Ti-6Al-4V substrate, showing the existence of Ti, Na, P, O, Al, and K, respectively, (**b**) EHA_Ti-6Al-4V, (i - vi) EDS mapping for EHA_Ti-6Al-4V EDS substrate, showing the presence of Ti, O, P, Na, Al, and Ca, respectively, (**c**) BHA_Ti-6Al-4V, (i - vi) EDS results for BHA_Ti-6Al-4V substrate, showing the presence of Ti, P, Al, Na, O, and K, respectively.

**Fig. 11:**
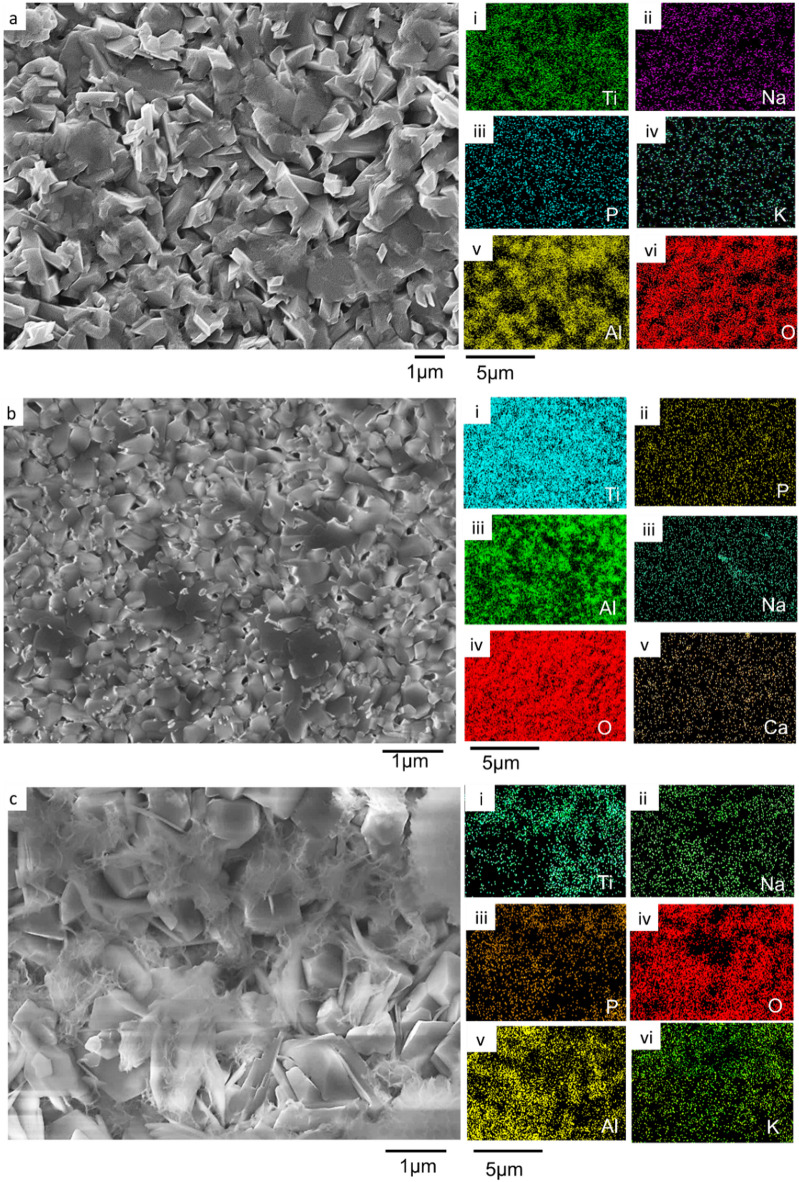
SEM micrograph of substrates immersed in SBF for week 1 (**a**) A_Ti-6Al-4V (i - vi) EDS mapping for A_Ti-6Al-4V substrate, showing the presence of Ti, Na, P, K, Al, and O, respectively, (**b**) EHA_Ti-6Al-4V (i - vi) EDS results for EHA_Ti-6Al-4V EDS substrate, showing the presence of Ti, P, Al, Na, O, and Ca, respectively, (**c**) BHA_Ti-6Al-4V, (i - vi) EDS results for BHA_Ti-6Al-4V substrate, showing the existence of Ti, Na, P, O, Al and K.

**Fig. 12:**
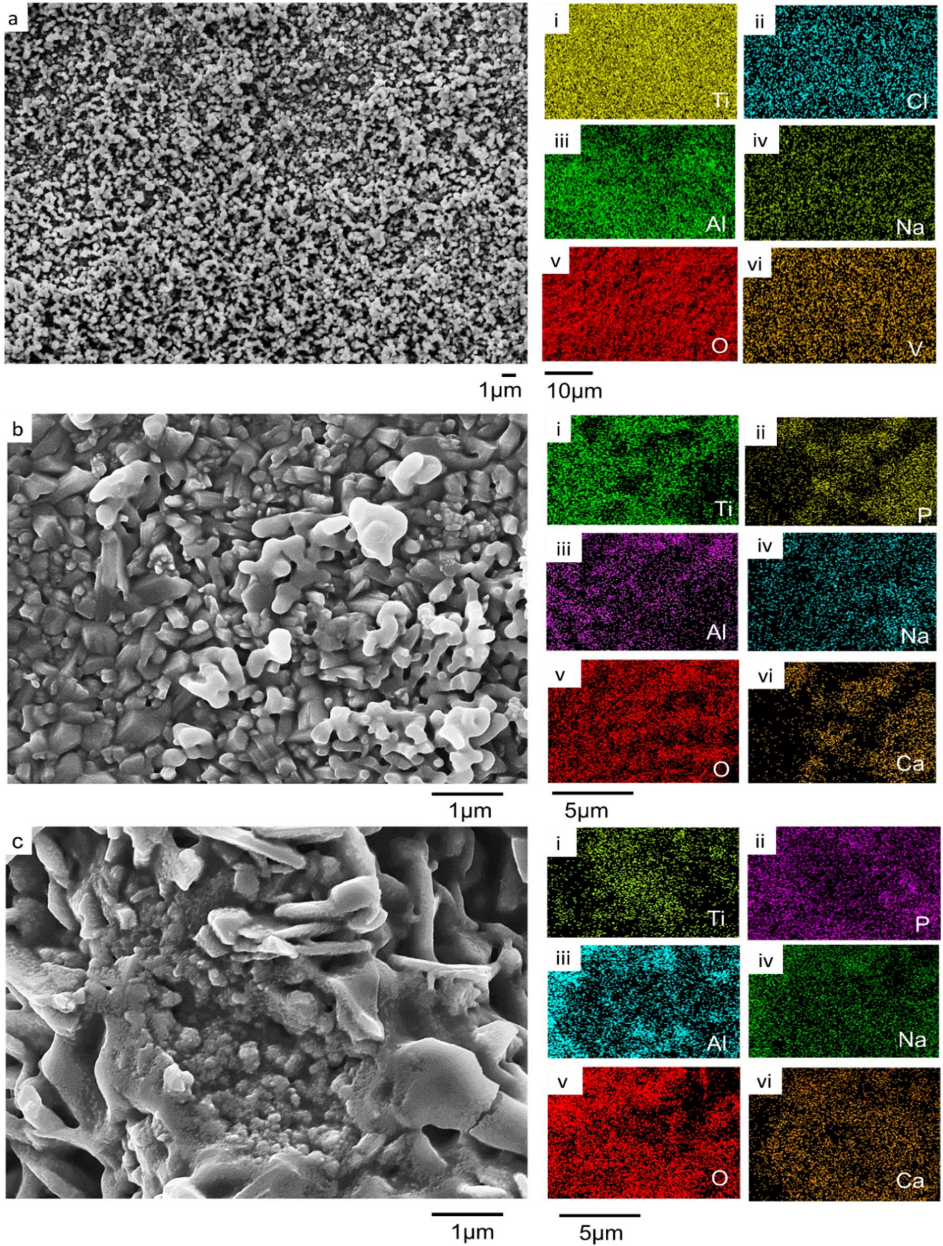
SEM micrograph of substrates immersed in SBF for week 2 (**a**) A_Ti-6Al-4V (i - vi) EDS results for A_Ti-6Al-4V substrate, showing the existence of Ti, Cl, Al, Na, O and V, (**b**) EHA_Ti-6Al-4V (i - vi) EDS results for EHA_Ti-6Al-4V EDS substrate, showing the presence of Ti, P, Al, Na, O, and Ca respectively, (**c**) BHA_Ti-6Al-4V, (i - vi) EDS results for BHA_Ti-6Al-4V substrate, showing the existence of Ti, P, Al, Na, O and Ca.

**Fig. 13:**
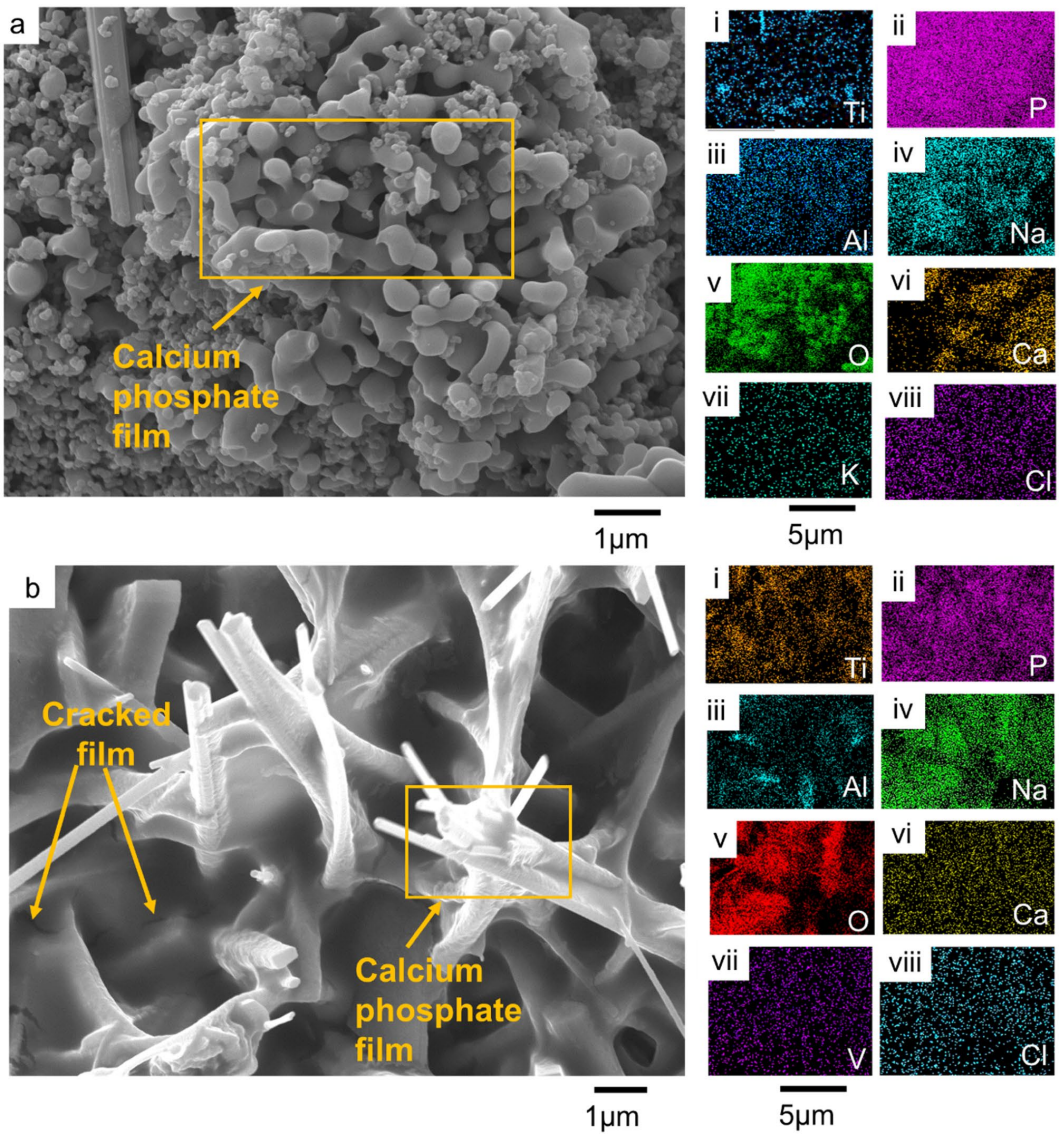
SEM micrograph of substrates immersed in SBF for week 3 (**a**) EHA_Ti-6Al-4V, (i - viii) EDS results for EHA_Ti-6Al-4V EDS substrate, showing the presence of Ti, P, Al, Na, O, Ca, K, and Cl respectively, (**b**) BHA_Ti-6Al-4V, (i - viii) EDS results for BHA_Ti-6Al-4V substrate, showing the existence of Ti, P, Al, Na, O, Ca, K, and Cl.

The immersion study in simulated body fluid (SBF) was conducted to evaluate the bioactivity of the coated samples by analyzing their ability to nucleate and grow calcium phosphate deposits. The SEM micrographs and compositional analysis of the substrates before exposing them to the SBF revealed the absence of P and Ca on the A_Ti-6Al-4V, while being present on the EHA_Ti-6Al-4V and BHA_Ti-6Al-4V, as shown in Fig. 10. The substrates immersed in the SBF were analyzed after 24 h, 1 week, 2 weeks and 3 weeks. The surface morphologies of the A_Ti-6Al-4V and the coated Ti-6Al-4V substrates after soaking in the SBF for the different durations are presented in Figs. 10, 11, 12 and 13.

After 24 h of soaking, the A_Ti-6Al-4V had no significant change in morphology. The EDS analysis, as shown in Fig. 10a (i – vi), however, showed additional elements like sodium (Na), Phosphorous (P), and Potassium (K) on the surface from the SBF. In contrast, after 24 h of soaking the EHA_Ti-6Al-4V in the SBF, flake-like structures formed, fused and covered the nanorods on the coated substrates, as shown in Fig. 10b. The EDS analysis showed a uniform spread of Ca and P across the surface, as illustrated in Fig. 10b (iii and vi). Similarly, after 24 h of soaking the BHA_Ti-6Al-4V in SBF, there was the formation of particulate structures on the microstructure of the coated substrate, as shown in Fig. 10c. The EDS analysis as shown in Fig. 10c (i - vi) showed the presence of Ca, P, K, and Na.

After week 1, visible changes in the morphology appeared as partial coverage, on some areas of the coating on the A_Ti-6Al-4V substrates, as presented in Fig. 11a. In addition to the inherent elements of the A_Ti-6Al-4V substrate, the EDS elemental mapping revealed the presence of Na, P, and K, as shown in Fig. 11a (i–vi). These ions may be present as surface-adsorbed ions associated with the SBF^62^. Notably, no Ca was detected on the surface, even as the duration of immersion increased. The bone-bonding capacity, typically characterized by the formation of Ca and P through immersion in SBF, was not observed for the Ti-6Al-4V, as observed in prior work^63,64^. After week 1 of immersing the EHA_Ti-6Al-4V substrates in the SBF, the surface appeared to have undergone significant changes, characterized by dense structures with some voids as shown in Fig. 11b. The EDS elemental mapping showed a uniform spread of Ca and P on the substrate as shown in Fig. 11b (i - v).

The BHA_Ti-6Al-4V substrate exhibited extensive nucleation and surface coverage by spherical calcium phosphate precipitates after one week of immersion in SBF, as observed in Fig. 11c. This surface deposition is indicative of the bioactivity and the formation of a bone-like apatite layer on the substrate, which is characteristic of HA-coated surfaces immersed in simulated body fluid^62,65,66^. The microstructural features of the BHA coating, including carbonate substitution and nanostructured roughness, likely enhanced ion exchange and promoted apatite nucleation.

At week 2, the A_Ti-6Al-4V substrates exhibited a spherical microstructural morphology, as shown in Fig. 12a. The EDS elemental mapping revealed the presence of Na and Cl from the SBF, in addition to the elements of the A_Ti-6Al-4V substrate, as depicted in Fig. 12a (i–vi). The EHA_Ti-6Al-4V substrates displayed a well-defined microstructure with a clear presence of Ca and P, confirmed by EDS elemental mapping, as shown in Fig. 12b. A related observation was made for the BHA_Ti-6Al-4V substrate at week 2 of immersion in the SBF, revealing full coverage, as shown in Fig. 12c. EDS elemental mapping confirmed the presence of Ca and P, as shown in Fig. 12c (i–vi).

By week 3, the morphology of the EHA_Ti-6Al-4V substrate became more diverse, revealing a combination of spherical and rod-like structures that were interconnected, as shown in Fig. 13a. EDS analysis revealed a uniform spread of Ca, P, and other elements from the SBF, as shown in Fig. 13a (i–viii). The BHA_Ti-6Al-4V substrate displayed full coverage with some cracks observed, as shown in Fig. 13b. EDS elemental mapping confirmed Ca, P, and other elements from the SBF on the substrate as illustrated in Fig. 13b (i–viii). The formation of calcium phosphate on the substrate indicated apatite formation, which signifies the bioactive nature and bone-bonding capacity of the HA-coated substrates, as observed in prior work^63,67^.

The studies presented in this table explore a range of hydroxyapatite (HA) deposition techniques, with varying degrees of consideration for sustainability. While some approaches, like the use of supersaturated solutions at low temperatures, hint at potentially more sustainable processing, the table largely lacks explicit discussion of environmental impact or resource efficiency. Furthermore, a notable gap exists in the reported key findings regarding the tribological properties of the deposited HA coatings. Information on wear resistance, friction coefficients, and long-term durability under mechanical stress is conspicuously absent across these diverse studies, representing a significant area for future research and understanding the practical applicability of these coatings, particularly in load-bearing biomedical implants.

These findings align with the tribological and mechanical performance results, reinforcing the multifunctional advantages of HA coating^26,68^. The ability of the HA-coated Ti-6Al-4V to facilitate calcium phosphate deposition in SBF suggests improved bioactivity, complementing the enhanced wear resistance and mechanical stability observed in previous analyses.

## Implications

The substrate of the annealed Ti-6Al-4V and Ti-6Al-4V coated by pack cementation with bovine bone and eggshell powder was characterized by the formation of titanium oxide and hydroxyapatite nanorods, these were confirmed by Raman, XRD, and EDS. The nano rod-like morphology was also confirmed by SEM and AFM analysis. The formation of titanium oxide on the annealed substrate has the potential to increase cell adhesion, proliferation, and mobility^63^. In terms of bone bonding capacity (i.e. bioactivity), Ti surfaces are required to be charged to successfully form apatite in the presence of body fluid^63^. Annealing Ti-6Al-4V at 900 °C does not result in a charged substrate, limiting the capacity to form apatite upon submerging in the SBF. This was observed from the absence of Ca from the elemental composition analysis after immersion into SBF. The two coated substrates on the other hand had visible Ca and P crystals forming on the surface. The formation of the apatite layers indicates their ability to bond in vivo through the apatite layer^63^. Previous studies have demonstrated the bone-bonding capacity of surface-modified titanium alloys with immersion in SBF^63,69^.

The hardness of the A_Ti-6Al-4V was less than the two coated substrates. EHA_Ti-6Al-4V recorded the highest hardness. Annealing and coating Ti-6Al-4V increased the CoF. The CoF is a significant parameter for implants. The coefficient of friction affects the tightening of dental implants and hence an increase in CoF results in the decreased tightening of the stresses on the maximum tightening moment^70^. The increase in the CoF observed in the annealed and coated substrates increases the initial stability of implant materials and subsequently improves osseointegration.

Coating Ti-6Al-4V with HA led to the development of an intermediate layer of titanium oxide between the Ti-6Al-4V substrate and the HA coating. This TiO_2_ layer was consistently present and contributed to the adhesion and stability of the HA coating on the Ti-6Al-4V substrate. This was evident even after the wear phenomenon of the HA from the coated substrate, where the TiO_2_ was revealed. This suggests a long-term advantage of using them as implants. The bone-bonding capacity of HA on the substrates initiates the growth of bone early enough. In the event of the wear of the HA from these substrates in service, the presence of titanium oxide will still be there with its advantage for implants. For implant materials, a decreased wear rate is desirable to prevent the effects of wear particles.

The SBF experiments ascertain the bioactivity of the substrate. The formation of the apatite layers on the HA-coated substrates from both bovine and eggshell sources indicates their ability to bond in vivo through the apatite layer^63,67^. The coated Ti-6Al-4V has been demonstrated to improve bioactivity with the formation of apatite upon immersion in SBF.

**Fig. 14:**
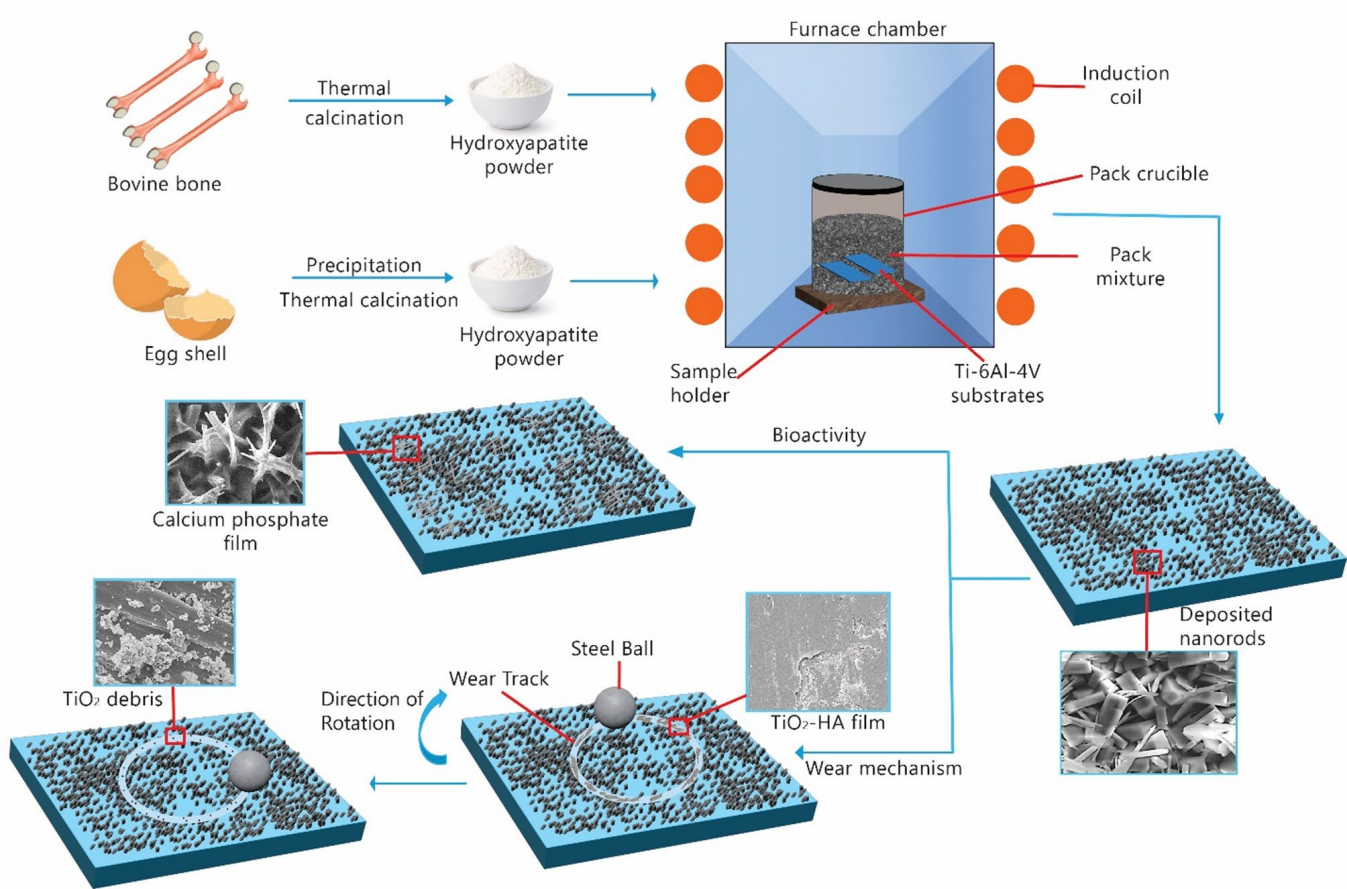
Schematic of HA Synthesis and Pack Cementation for Coating HA Nanorods onto Ti-6Al-4V: Bioactive Substrate Formation with Enhanced Tribological Properties.

The schematic in Fig. 14 illustrates the novel process developed for creating and applying hydroxyapatite nanorod coatings on Ti-6Al-4V substrates using pack cementation. This technique resulted in bioactive Ti-6Al-4V substrates with markedly improved tribological properties, essential for enhancing wear resistance in dental implants. The successful deposition of HA nanorods represents a notable advancement in surface engineering, potentially enhancing osseointegration and minimizing wear-related failures in dental implants. Figure 14 emphasizes the practical applications of this coating method in developing cutting-edge biomaterials that optimize both the biological and mechanical performance of implantable dental materials.

## Conclusion

Based on the results and discussion, the conclusions are:

- Surface modification of Ti-6Al-4V through annealing and HA coating significantly enhances its suitability for dental and orthopaedic implants.
- These treatments introduce nanostructured roughness, improving cell–surface interactions critical for implant integration.
- While both annealed and HA-coated surfaces exhibit nanostructures, the HA-coated substrates provide superior surface chemistry that closely mimics natural bone, crucial for effective osseointegration.
- The annealed substrates developed a rutile-phase TiO₂ morphology, enhancing surface reactivity, whereas the HA nanorods further optimized the surface to promote bone-like apatite formation, confirmed through simulated body fluid (SBF) testing.

Tribological performance showed that:

- BHA-coated substrates had a coefficient of friction (CoF) of 0.70, wear rate of 0.020 mm³/Nm, and hardness of 1.08 GPa.
- EHA-coated substrates had a CoF of 0.79, wear rate of 0.030 mm³/Nm, and hardness of 1.82 GPa.
- These values demonstrate high wear resistance and mechanical stability.
- A dual-layer mechanism emerged from the pack cementation process:
- A base layer of TiO₂ formed on Ti-6Al-4V.
- A top layer of HA was deposited, which gradually wears away, exposing TiO₂ that maintains reactivity and supports long-term bone integration.
- The transition from HA to TiO_2_ ensures early-stage bioactivity for osseointegration and long-term mechanical durability of the implant.

Overall, HA-coated Ti-6Al-4V fabricated via pack cementation provides a cost-effective, bioactive, and wear-resistant solution for dental and orthopaedic applications.

## Data Availability

Data is provided within the manuscript. Data will be made available upon reasonable request from the corresponding author (soboyew@sunypoly.edu).
